# Bcl2 inhibits recruitment of Mre11 complex to DNA double-strand breaks in response to high-linear energy transfer radiation

**DOI:** 10.1093/nar/gku1358

**Published:** 2015-01-07

**Authors:** Maohua Xie, Dongkyoo Park, Shuo You, Rui Li, Taofeek K. Owonikoko, Ya Wang, Paul W. Doetsch, Xingming Deng

**Affiliations:** 1 Departments of Radiation Oncology, Division of Cancer Biology, Emory University School of Medicine and Winship Cancer Institute of Emory University, Atlanta, Georgia 30322, USA; 2 Hematology and Medical Oncology, Emory University School of Medicine and Winship Cancer Institute of Emory University, Atlanta, Georgia 30322, USA; 3 Biochemistry, Emory University School of Medicine and Winship Cancer Institute of Emory University, Atlanta, Georgia 30322, USA

## Abstract

High-linear energy transfer ionizing radiation, derived from high charge (Z) and energy (E) (HZE) particles, induces clustered/complex DNA double-strand breaks (DSBs) that include small DNA fragments, which are not repaired by the non-homologous end-joining (NHEJ) pathway. The homologous recombination (HR) DNA repair pathway plays a major role in repairing DSBs induced by HZE particles. The Mre11 complex (Mre11/Rad50/NBS1)-mediated resection of DSB ends is a required step in preparing for DSB repair via the HR DNA repair pathway. Here we found that expression of Bcl2 results in decreased HR activity and retards the repair of DSBs induced by HZE particles (i.e. ^56^iron and ^28^silicon) by inhibiting Mre11 complex activity. Exposure of cells to ^56^iron or ^28^silicon promotes Bcl2 to interact with Mre11 via the BH1 and BH4 domains. Purified Bcl2 protein directly suppresses Mre11 complex-mediated DNA resection *in vitro*. Expression of Bcl2 reduces the ability of Mre11 to bind DNA following exposure of cells to HZE particles. Our findings suggest that, after cellular exposure to HZE particles, Bcl2 may inhibit Mre11 complex-mediated DNA resection leading to suppression of the HR-mediated DSB repair in surviving cells, which may potentially contribute to tumor development.

## INTRODUCTION

Terrestrial radiation is largely characterized by low-linear energy transfer (low-LET) radiation (i.e. X-, β- or γ-rays) while space radiation is characterized by high-linear energy transfer ionizing radiation (high-LET IR) (1). High-LET IR is induced by high-charge (HZE) particles (a component in space radiation) or high energy ions. High-LET IR induces clustered/complex DNA damage, including double-strand DNA breaks (DSBs) and non-DSB oxidative clustered DNA lesions (2–5). Clustered DSBs may be converted into replicative DSBs in S phase. These replicative breaks as well as persistent frank DSBs left unrepaired by nonhomologous end-joining (NHEJ) may be repaired by homologous recombination (HR) (6). It has recently been reported that high-LET IR derived from ^56^iron (^56^Fe) ion can inhibit the Ku-dependent main NHEJ pathway by the generation of small fragments of DNA DSBs (<40 bp) but cannot inhibit the HR repair pathway (2), suggesting that the processing of complex DSBs induced by high-LET IR involves an interplay between NHEJ and HR. Since high-LET IR-induced small fragments of DNA DSBs did not affect the binding efficiency of Mre11 with DSBs (3), the Mre11-dependent HR repair pathway may play a more important role than Ku-dependent NHEJ in the repair of high-LET IR-induced DSBs. Thus, DNA end resection may be the first and essential step in repairing high-LET IR-induced DSBs.

Mre11 nuclease forms the core of the Mre11-Rad50-NBS1 (MRN) complex, which has been implicated in the detection of DSBs, DNA end resection, recombination, S or G2/M checkpoint control and promotion of DSB repair by HR (7,8). The HR pathway starts with resection of DSBs, which requires the high-affinity association of the MRN complex with broken DNA ends and the Mre11 subunit endonuclease and exonuclease activities (9,10). Processing of the ends by DNA resection yields 3′ single-stranded DNA (ssDNA) tails or overhangs, which are required for the Rad51-initiated HR repair pathway (11). DNA-end resection, an evolutionarily conserved process that generates long stretches of single-stranded DNA, plays a critical role in pathway choice, as it commits cells to HR, while, at the same time, suppressing NHEJ (12).

Bcl2 not only negatively regulates NHEJ but also the HR pathway during the repair of DSBs (13,14). Bcl2 can specifically suppress the Rad51-mediated HR pathway (14). It remains unknown whether and how Bcl2 regulates Mre11 complex-mediated DNA resection in the repair of high-LET IR-induced clustered DSBs. We report here that Bcl2 suppresses DNA resection by direct interaction with Mre11 following high-LET IR exposure leading to suppression of the HR repair pathway. These results have important implications for the biological endpoints of human exposure to high-LET IR.

## MATERIALS AND METHODS

### Materials

Bcl2, Rad50 and Ku70 antibodies were purchased from Santa Cruz Biotechnology (Santa Cruz, CA, USA). NBS1 antibody was purchased from Novus Biologicals (Littleton, CO, USA). Mre11 antibody was purchased from Cell Signaling Technology, Inc. (Danvers, MA, USA). γH2AX antibody was purchased from EMD Millipore (Billerica, MA, USA). 53BP1 antibody was purchased from BD Transduction Laboratories. Purified, recombinant wild-type (WT) and Bcl2 homology (BH) deletion Bcl2 mutant proteins were obtained from ProteinX Lab (San Diego, CA, USA). Alexa Fluor 488 (green) goat anti-mouse, Alexa Fluor (red) 555 goat anti-rabbit, SYBR Gold Nucleic Acid Gel Stain and Sf9 cells were purchased from Invitrogen Life Technologies Inc. (Carlsbad, CA, USA). Anti-FlagM2 affinity gel and Flag peptide were purchased from Sigma (St Louis, MO, USA). Hiprep^™^ 16/60 Sepharcryl™ S-100 HR and HiTrap Q Sepharose FF column were purchased from GE Healthcare (Waukesha, WI). [γ-^32^P]-ATP was obtained from PerkinElmer (Waltham, MA, USA). pCBASce and DR-GFP (green fluorescent protein) constructs were purchased from Addgene (Cambridge, MA, USA). Purified recombinant Ku70 was purchased from Trevigen Inc. (Gaithersburg, MD, USA). Nucleofector™ kit was purchased from Lonza Group Ltd (Houston, TX, USA). The PhiX174 circular single-stranded virion DNA substrate was purchased from New England Biolabs (Ipswich, MA, USA). Double-strand DNA (dsDNA) oligonucleotides (TP74 and TP124) were obtained from Integrated DNA Technologies (Coralville, IA, USA). All of the reagents used were obtained from commercial sources unless otherwise stated.

### Cell lines, plasmids, transfections and radiation

Normal human bronchial epithelial cell line (BEAS-2B), H1299 and H460 lung cancer cell lines were obtained from the American Type Culture Collection (ATCC, Manassas, VA, USA). BEAS-2B cells were cultured in DMEM/F-12 medium supplemented with 10% fetal bovine serum (FBS). H1299 and H460 cells were cultured in RPMI 1640 medium supplemented with 5% FBS and 5% bovine serum. These cell lines were employed for the described experiments without further authentication. WT and various BH deletion mutants (i.e. ΔBH1, ΔBH2, ΔBH3 and ΔBH4) were created and stably expressed in H1299 or BEAS-2B cells as previously described (13). The expression levels of exogenous Bcl2 were analyzed by western blot analysis, and three separate clones expressing similar amounts of exogenous Bcl2 were selected for further analysis. High-LET IR derived from ^56^Iron (^56^Fe) or ^28^Silicon (^28^Si) was performed under the Alternating Gradient Synchrotron Booster at the Brookhaven National Laboratory in New York. Cells were exposed to 1 Gy (dose rate: 0.5 Gy/min) of ^56^Fe (600 MeV/u) or ^28^Si (300 MeV/u), followed further analysis. Low-LET IR was performed using an X-ray machine (X-RAD 320, North Branford, CT, USA) at Emory University.

### Preparation of cell lysate and immunoprecipitation

Cells were washed with 1 × phosphate buffered saline (PBS) and resuspended in ice-cold 1% CHAPS lysis buffer (1% CHAPS, 50 mM Tris [pH 7.6], 120 mM NaCl, 1 mM ethylenediaminetetraacetic acid (EDTA), 1 mM Na_3_VO_4_, 50 mM NaF and 1 mM β-mercaptoethanol) with a cocktail of protease inhibitors (EMD Biosciences). Cells were lysed by sonication and centrifuged at 14 000 × *g* for 10 min at 4°C. The resulting supernatant was collected as the total cell lysate. For immunoprecipitation, 1 mg of whole-cell extract was applied to 30 μl of protein A Sepharose slurry (Invitrogen) together with 5 μg of the specified antibodies or agarose-conjugated Bcl2 (Santa Cruz, CA, USA). After overnight incubation at 4°C and stringency washes, elution was carried out by boiling in Laemmli buffer.

### Immunofluorescence

Cells were grown on LabTek 8-well chamber slides, then fixed with methanol and acetone (1:1) for 5 min at −20°C. After blocking with 10% normal goat serum for 20 min at room temperature, chamber slides were incubated with mouse γ-H2AX or rabbit 53BP1 primary antibodies overnight at 4°C. After washing with PBS, the cells were incubated with Alexa 488 (green)-conjugated anti-mouse or Alexa 555 (red)-conjugated anti-rabbit secondary antibodies or DAPI for 60 min at room temperature. Cells were washed with PBS and observed under a fluorescent confocal microscope (Zeiss, Sweden). γ-H2AX or 53BP1 foci tracks were quantified as described (4,6,15). The number of tracks in each nucleus was manually counted from 100 nuclei for each sample. The percent tracks of γ-H2AX or 53BP1 foci remaining at 72 h was normalized to the track number at 0 h time point. Error bars represent ± SD of three repeated determinations.

### Homologous recombination assay

HR was carried out as previously described (16). The reporter used in this study consists of two differentially mutated GFP genes oriented as direct repeats. pCBASce and DR-GFP were co-transfected into BEAS-2B, H1299 or H460 cells by electroporation. After 48 h, cells were harvested and subjected to flow cytometry analysis to determine percentages of GFP-positive cells. Expression of I-SceI endonuclease generates a site-specific DSB between the mutated GFP genes. Once the DSB in GFP is repaired by HR, a functional GFP gene is generated. Samples were analyzed in a BD Biosciences FACScan and data were processed using FlowJo software.

### Production and purification of MRN proteins

Baculoviruses with Flag-Mre11, Flag-NBS1 or His_6_-Rad50 were a kind gift from Dr TT Paul (University of Texas at Austin) (17,18). The MRN complex as well as the individual MRN subunits were produced in Sf9 cells infected with appropriate baculoviruses. After infection 72 h, cells harvested were suspended in buffer A [50 mM Tris (pH 7.4), 0.15 M NaCl, 1% (v/v)Tween 20, 0.3%NP-40,10% (v/v) glycerol, 2 mM Dl-dithiothreitol (DTT)] supplemented with 2 mM phenylmethylsulfonylfluoride and disrupted by sonication. The cell extract was centrifuged at 50000 × *g* for 1 h and loaded on a 1-ml column (Sigma, St. Louis, MO, USA) equilibrated with buffer A containing anti-FLAG M2 antibody conjugated to agarose beads. Bound proteins were eluted using with 2 ml buffer A containing 100 μg/ml Flag peptide on an AKTA Prime Plus system (GE Healthcare, Waukesha, WI, USA). Fractions containing MRN proteins were collected, dialyzed against buffer B (20 mM Tris, pH 8.0, 100 mM NaCl, 10% (v/v) glycerol and 1 mM DTT) and loaded onto a 1-ml HiTrap Q Sepharose FF column (GE Healthcare, Waukesha, WI). Bound proteins were eluted with a linear concentration gradient of NaCl (i.e. 50 and 500 mM) in 10 ml buffer B. RAD50 was purified using 5-ml HiTrap Ni2+-Sepharose column equilibrated with buffer A containing 20 mM imidazole. Bound proteins were eluted with a linear concentration gradient of imidazole (i.e. 50 and 350 mM) in 50 ml buffer A. Fractions containing Rad50 protein were pooled, dialyzed against buffer B (20 mM Tris-HCl, pH 8.0, 100 mM NaCl, 10% (v/v) glycerol and 1 mM DTT) and loaded onto a 1-ml HiTrap Q Sepharose FF column. Bound proteins were eluted with a linear concentration gradient of NaCl (i.e. 50 and 500 mM) in 12 ml buffer B. The NBS1 protein was purified only using HiTrap Q Sepharose FF column as it was expressed without a tag. Purified proteins were stored at −80°C. The concentration of purified proteins was determined by Bradford assay. The values obtained by Bradford assay were divided by the predicted molecular mass of the appropriate protein to calculate its molar concentration.

### DNA resection assay

First, Mre11 complex endonuclease activity was measured using PhiX174 circular single-stranded virion DNA substrate as described previously (11,19,20). One nanomolar DNA substrate was incubated with purified 10 nM of Mre11, NBS1 or RAD50 or MRN complex in the absence or presence of purified Bcl2 in 20 μl reaction buffer (30 mM potassium-MOPS (pH 7.0), 25 mM KCl, 1 mM DTT, 5 mM MnCl_2_, 2 mM ATP and 50 μg/ml acetylated bovine serum albumin (BSA)) at 37°C for 3 h. Reactions were terminated by adding 1/10 volume of stop solution (3% sodium dodecylsulphate (SDS), 50 mM EDTA) and proteinase K (Thermo Fisher Scientific, Waltham, MA, USA) to a final concentration of 1 mg/ml and incubating at 37°C for 10 min. The reaction products were run on a 0.8% agarose gel (1 × Tris-acetate-EDTA) for 90 min at 100 mA. DNA was stained with SYBR Gold Nucleic Acid Gel Stain (Invitrogen Carlsbad, CA, USA) for 20 min and visualized with a Typhoon 9200 scanner (GE healthcare, Waukesha, WI). Second, effect of Bcl2 on exonuclease activity of Mre11 complex was measured using double-stranded (ds) substrate as described previously (20–22). Double-stranded (ds) DNA substrate was generated by annealing purified oligonucleotides TP74 (CTG CAG GGT TTT TGT TCC AGT CTG TAG CAC TGT GTA AGA CAG GCC A) to TP124 (CAT CTG GCC TGT CTT ACA CAG TGC TAC AGA CTG GAA CAA AAA CCC TGC AG), with TP74 labeled with [^32^P] at the 5′ end. Fifty nanomolar DNA substrates were incubated with 10 nM of purified Mre11, NBS1 or RAD50 or MRN complex in the absence or presence of purified Bcl2 in 20 μl exonuclease buffer (25 mM morpholinepropanesulfonic acid, pH 7.0, 60 mM KCl, 0.2% Tween 20, 2 mM dithiothreitol, 2 mM ATP, 5 mM MnCl2) at 37°C for 30 min. Reactions were terminated by adding 1/10 volume of stop solution (3% SDS, 50 mM EDTA) and proteinase K to a final concentration of 1mg/ml and incubating at 37°C for 15 min. Reactions were loaded on an 15% acrylamide/urea gel, run at 75 W for 120 min, dried onto Whatman filter paper and visualized with a Typhoon 9200 scanner (GE healthcare, Waukesha, WI).

### Electrophoretic mobility shift assay (EMSA)

Double-strand DNA (dsDNA) end binding of the proteins was assessed by EMSA as described previously (22,23). Oligonucleotides were chemically synthesized and purified by HPLC (IDT, Coralville, Iowa). The sequences of oligonucleotides were: TP423: 5′ -CTG CAG GGT TTT TGT TCC AGT CTG TAG CAC TGT GTA AGA CAG GCC AGA TC-3′; and TP424: 5′-CAC AGT GCT ACA GAC TGG AAC AAA AAC CCT GCA GTA CTC TAC TCA TCT C- 3′. First, the 5′ end of TP423 (0.4nM) was labeled with [γ-^32^P]-ATP and polynucleotide kinase (New England Biolabs, Ipswich, MA, USA). Non-labeled [γ-^32^P]-ATP was removed using G-25 Spin Columns. The 3′ overhanging DNA duplexes were produced by annealing of TP423 and TP424 oligonucleotides. The 3′ overhanging DNA duplexes (10 pM) were incubated with Mre11 (10 nM) in the absence or presence of increasing concentrations of Bcl2 protein in binding buffer [25 mM MOPS, pH 7.0, 60 mM KCl, 0.2% Tween, 2 mM DTT, 1 mM Mg(CH3COO)_2_] at 37°C for 15 min. Gel loading buffer [2.5 ml, 250mM Tris–HCl (pH 7.5), 0.2% bromphenol blue and 40% glycerol] was then added to the reaction. The reaction products were separated on a 5% non-denaturing polyacrylamide gel run at 130V and 4°C in Tris borate/EDTA buffer for 2 h. The signals of labeled DNA fragments were detected using a PhosphoImager.

### Chromatin immunoprecipitation (ChIP)

DNA in Ku or MRE11 complexes was measured as previously described (24). Briefly, 10^7^ cells were treated with 1 Gy of IR or ^56^Fe, ^37^Si. Protein–DNA complexes in treated cells were cross-linked with 1% formaldehyde. Cells were resuspended with buffer A (50 mM Tris, pH 8.1, 1% SDS; and 10 mM EDTA), followed by sonication. The samples were immunoprecipitated using Ku70 or Mre11 antibody, respectively in buffer B (16.7 mM Tris,pH 8.1, 1% SDS; 1% Triton X-100; 1.2 mM EDTA; 167 mM NaCl). The immunoprecipitation complexes were washed one time with buffer C (20 mM Tris, pH8.1, 0.1% SDS, 1% Triton X-100, 2 mM EDTA and 150 mM NaCl), buffer D (500 mM NaCl) and buffer E (10 mM Tris, pH 8.1, 25% LiCl, 1% NP40, 1% deoxycholic acid and 1 mM EDTA), then two times with Tris–EDTA buffer (pH 8.0). DNA in Ku70–DNA or Mre11–DNA complexes was labeled with [γ-^32^P]ATP by incubation with T4 polynucleotide kinase in kinase buffer at 37°C for 45 min and then at 65°C for 10 min. The samples were then washed four times with TE buffer (pH 8.0) and incubated in TE buffer containing 1 mg/ml protease K (Thermo Fisher Scientific, Waltham, MA, USA) at 37°C for 2 h. The samples were separated on a 5% non-denaturing polyacrylamide gel. DNA signals were detected and analyzed using a PhosphoImager with ImageQuant software (GE health, Waukesha, WI).

### Bcl2 silencing

Bcl2 shRNA and control shRNA were obtained from Santa Cruz Biotechnology (Santa Cruz, CA, USA). Hairpin sequence of Bcl2 shRNA: GAT CCG TGT GGA TGA CTG AGT ACC TGA TTC AAG AGA TCA GGG ACT CAG TCA TCC ACA TTT TTG. Hairpin sequence of control shRNA: GAT CCG GAA CGG CAT CAA GGT GAA CTT CAA GA GAG TTC ACC TTG ATG CCG TTC TTT TTG. For pseudovirus production, Bcl2 shRNA or control shRNA was cotransfected into 293FT cells with a lentivirus packaging plasmid mixture (System Biosciences, CA, USA) using the NanoJuice transfection kit (EMD Chemical, Inc.) as described (25). After 48 h, the virus-containing media were harvested by centrifugation at 20 000 × *g*. H460 cells were infected with the virus-containing media in the presence of polybrene (8 μg/ml) for 24 h. Stable positive clones were selected using 1 μg/ml puromycin. Speciﬁc silencing of the targeted Bcl2 gene was conﬁrmed by at least three independent experiments.

### Statistical analysis

Significant differences between every two groups were analyzed using Fisher's exact test or two-sided unpaired Student's *t*-test. A *P*-value < 0.05 was considered statistically significant.

## RESULTS

### High-LET IR derived from ^56^Fe or ^37^Si particles induces γH2AX and 53BP1 foci along ion tracks

**Figure 1.**
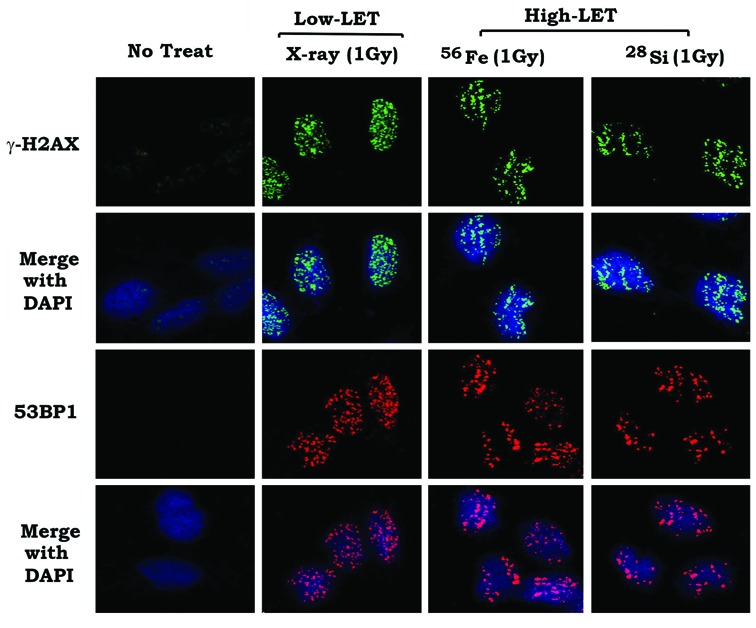
Comparison of low-LET and high-LET IR induces DNA double-strand breaks. BEAS-2B cells were exposed to 1 Gy of low-LET IR (i.e. X-ray) or high-LET IR (i.e. ^56^Fe or ^37^Si). γ-H2AX or 53BP1 foci were analyzed by immunostaining.

To compare high-LET and low-LET IR-induced DSBs, normal human bronchial epithelial (BEAS-2B) cells were exposed to 1 Gy of X-ray (i.e. low-LET IR) or ^56^Fe or ^28^Si ions (i.e. high-LET-IR). DSBs were analyzed by immunostaining using γH2AX or 53BP1 antibody, respectively. Track structure is known to be a critical determinant of the biological effectiveness of charged-particle radiation (4,26). As expected, high-LET IR derived from HZE ^56^Fe or ^28^Si particles induced γH2AX and 53BP1 foci along the ion track (i.e. clustered DSBs) while low-LET IR induced DSB foci with sparse ionization, and DNA breaks were scattered more evenly throughout the nucleus (Figure 1). High-LET IR-induced small fragments of DNA DSBs have been reported to inhibit the Ku-dependent NHEJ pathway but does not affect the HR pathway, indicating that the HR pathway plays a major role in repairing DSBs induced by high-LET IR (3).

### Bcl2 suppresses the repair of high-LET IR-induced DNA damage

**Figure 2.**
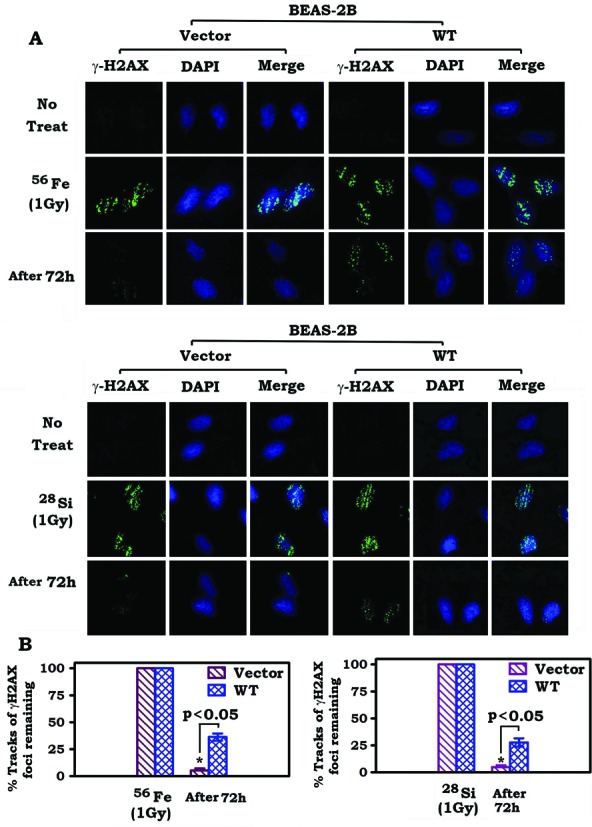
Expression of Bcl2 retards the repair of high-LET IR-induced clustered DSBs. (**A**) BEAS-2B cells overexpressing Bcl2 or vector-only control were exposed to 1 Gy of ^56^Fe or ^28^Si. Cells were harvested after 72 h. DSB tracks were measured by immunostaining using γ-H2AX antibody. (**B**) γ-H2AX foci tracks were quantified as described in ‘Materials and Methods’ section.

We previously reported that Bcl2 can retard DSB repair following low-LET IR exposure by inhibition of the NHEJ pathway (13). To further address whether Bcl2 affects the repair of high-LET IR-induced DSBs, DSBs were analyzed by measurement of γH2AX or 53BP1 foci tracks as described (15,27,28). As shown in Figure 2 and Supplementary Figure S1, 1 Gy of high-LET IR derived from ^56^Fe or ^28^Si particles resulted in robust induction of tracks of γH2AX or 53BP1 foci in both vector-only control and Bcl2 overexpressing cells. However, the percentage of tracks of γ-H2AX or 53BP1 foci remaining was significantly higher in BEAS-2B cells expressing WT Bcl2 compared to vector-only control cells. Similar results were also obtained in H1299 cells following exposure to ^56^Fe or ^28^Si particles (Supplementary Figures S2 and S3). These findings indicate that Bcl2 can also retard the repair of high-LET IR-induced DSBs in a mechanism involving suppression of the HR pathway.

### Expression of Bcl2 results in decreased HR activity and suppression of high-LET IR-induced MRE11/ DNA binding

**Figure 3.**
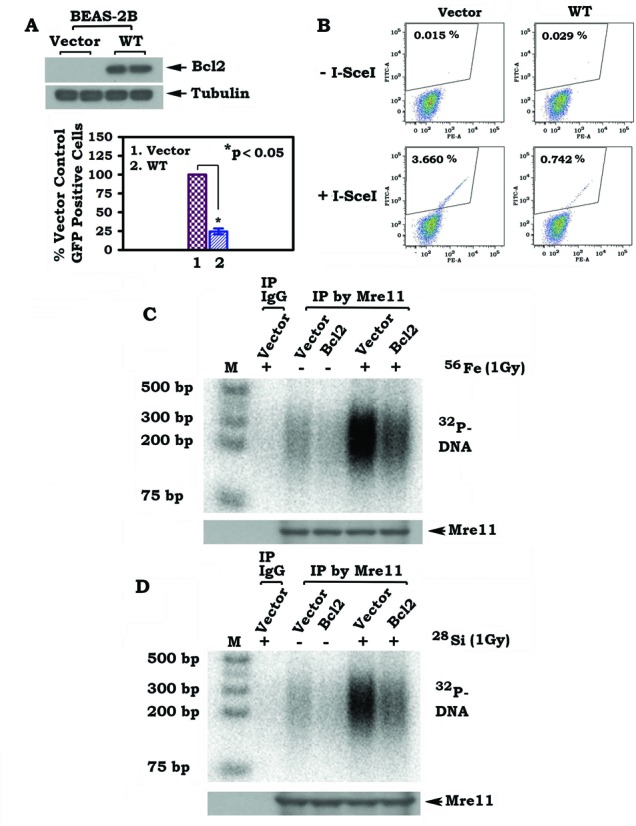
Expression of Bcl2 suppresses homologous recombination (HR) and Mre11/DNA binding. (**A**) Bcl2 was overexpressed in BEAS-2B cells and expression levels were analyzed by western blot. (**B**) HR activity was measured using pCBASce and DR-GFP system in BEAS-2B cells overexpressing Bcl2 or vector-only control as described in ‘Materials and Methods’ section. Percentage of GFP-positive cells was analyzed by flow cytometry. (**C**) and (**D**) BEAS-2B cells overexpressing Bcl2 or vector-only control were exposed to 1 Gy of ^56^Fe or ^28^Si. Mre11/DNA binding was analyzed by ChIP assay.

Bcl2 has previously been demonstrated to inhibit both NHEJ and HR DNA repair pathways (13,14,29). To test whether Bcl2 regulates HR activity in BEAS-2B cells, a GFP reporter-based approach was employed for measurement of HR activity as described in ‘Materials and Methods’ section. We observed that expression of Bcl2 resulted in a 5-fold reduction of HR in BEAS-2B cells (Figure 3A and B). Similar results were also observed in H1299 lung cancer cells (Supplementary Figure S4). These data support and extend previous reports (14,29). To determine the cellular Bcl2 level that is high enough to have an effect on HR following HZE particle exposure, a dose-response experiment was performed using increasing amounts of Bcl2 cDNA for transfection into BEAS-2B cells. BEAS-2B cells expressing gradually increasing levels of Bcl2 were analyzed for HR. Results showed that Bcl2 inhibition of HR occurs in a dose-dependent manner (Supplementary Figure S5). Bcl2 expression resulting from transfection using 1 μg or above of Bcl2/pCIneo constructs caused significant inhibition of HR activity (i.e. *P* < 0.05; Supplementary Figure S5), indicating this level of Bcl2 expression (lane 4) may be high enough to have a significant effect on HR. To test whether Bcl2 affects high-LET IR-induced Mre11/DNA binding, BEAS-2B cells expressing Bcl2 or vector-only control were exposed to 1 Gy of high-LET IR from ^56^Fe or ^28^Si particles, followed by ChIP employing Mre11 antibody. Expression of Bcl2 reduced ^56^Fe or ^28^Si particle-induced Mre11/DNA binding (Figure 3C and D), suggesting that Bcl2 may negatively affect HR-mediated DNA repair after high-LET IR exposure.

### High-LET IR stimulates Bcl2 accumulation in the nucleus

Bcl2 is known to be primarily localized in the outer mitochondrial membranes with minor expression in the nucleus (30). We previously discovered that low-LET IR stimulates Bcl2 accumulation in the nucleus (13). To test whether high-LET IR has a similar effect on Bcl2 localization, BEAS-2B cells expressing WT Bcl2 were treated with increasing doses (i.e. 0.1–1 Gy) of high-LET IR derived from ^56^Fe or ^28^Si particles, followed by subcellular fractionation as previously described (13). Results revealed that high-LET IR enhanced Bcl2 expression in the nucleus in a dose-dependent manner but did not affect Bcl2 expression in the mitochondrial fraction (Supplementary Figure S6). It is possible that high-LET radiation-induced nuclear localization of Bcl2 may render Bcl2 able to interact with Mre11 in the nucleus, leading to suppression of HR activity.

### Bcl2 directly interacts with Mre11 via its BH1 and BH4 domains

**Figure 4.**
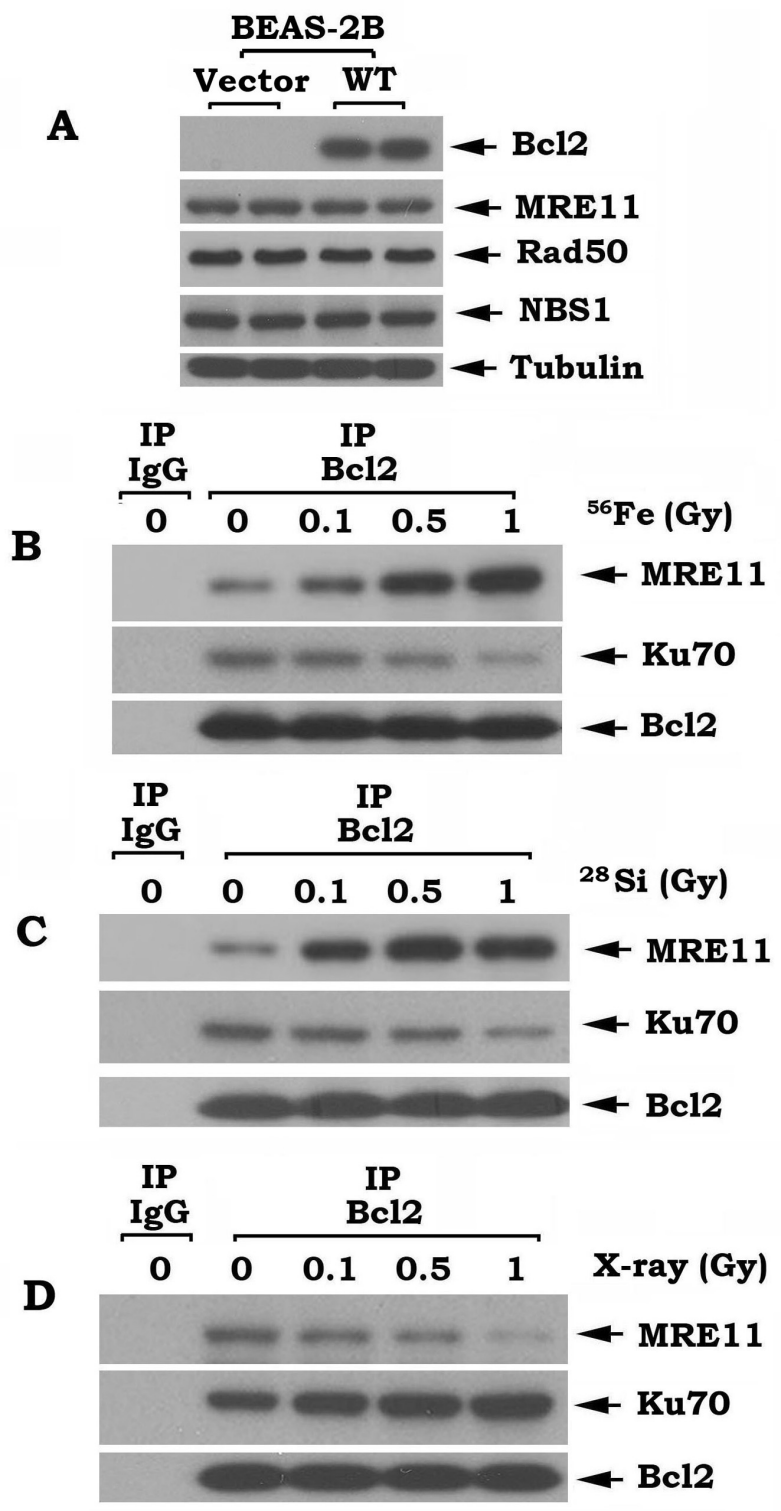
Effect of high-LET and low-LET IR on Bcl2/Mre11 and Bcl2/Ku70 binding. (**A**) Western blot analysis of Bcl2, Mre11, Rad50 and NBS1 in BEAS-2B cells overexpressing Bcl2 or vector-only control. (**B**), (**C**) and (**D**) BEAS-2B cells overexpressing Bcl2 were treated with increasing doses of ^56^Fe, ^28^Si or X-ray. The co-IP experiments were performed using agarose-conjugated Bcl2 antibody. Bcl2-associated Mre11 or Ku70 were analyzed by western blot.

**Figure 5.**
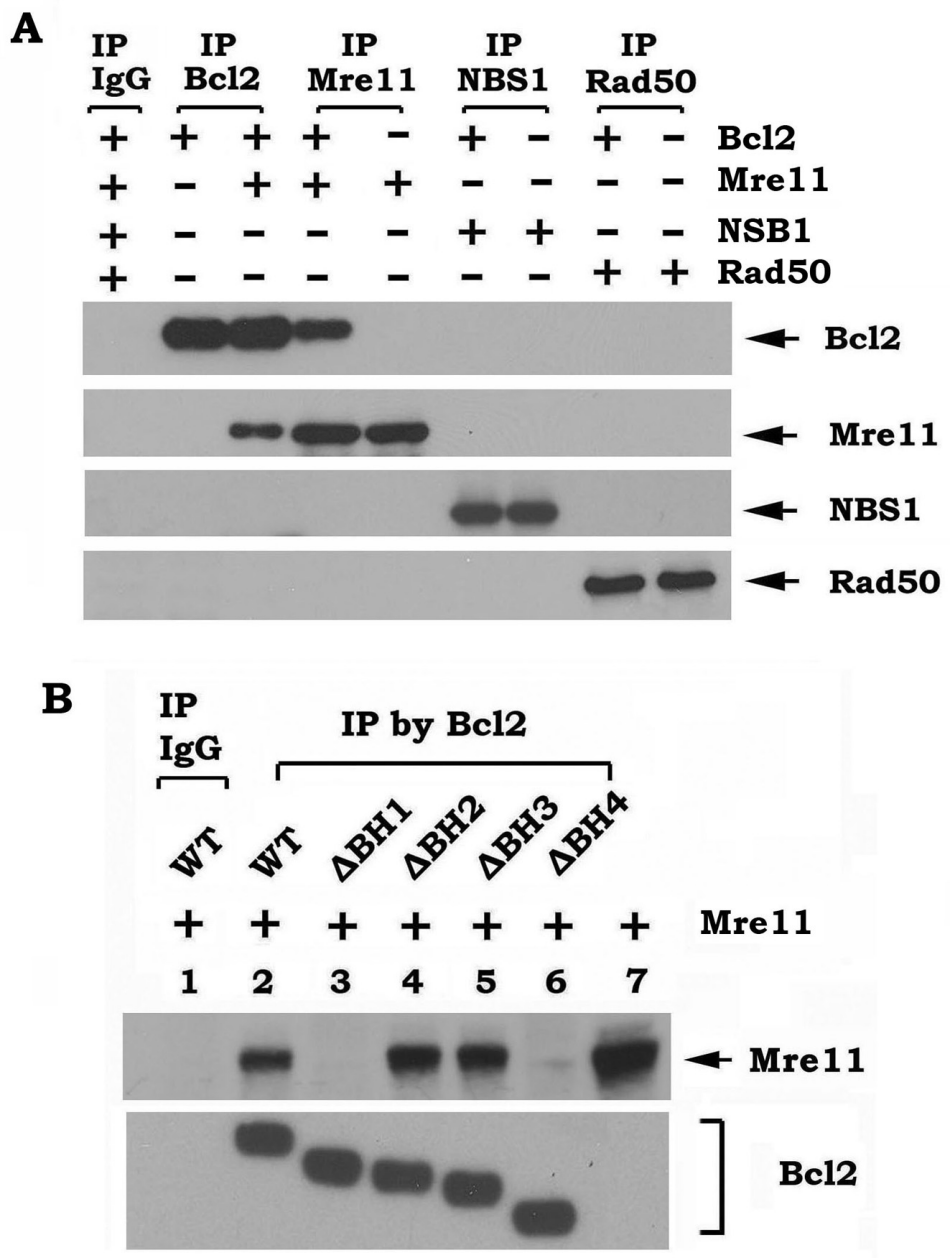
Bcl2 directly interacts with Mre11 via its BH1 and BH4 domain. (**A**) Purified recombinant WT Bcl2 (20 ng/ml) was incubated with purified Mre11, or NBS1 or RAD50 (20 ng/ml) in 1% CHAPS lysis buffer at 4°C for 2 h. Co-IP experiments were performed using Bcl2, MRE11 or NBS1 or RAD50 antibody, respectively. Bcl2, Mre11, NBS1 and RAD50 were analyzed by western blot analysis. (**B**) Purified WT or each BH deletion Bcl2 mutant protein (20 ng/ml) was incubated with 20 ng/ml of purified Mre11 in 1% CHAPS lysis buffer at 4°C for 2 h. Co-IP was carried out using agarose-conjugated Bcl2 antibody. Bcl2 and Mre11 were analyzed by western blot.

After DSBs occur, the Mre11 protein is critical in mediating DNA end resection and the HR repair pathway (3,31–34). Based on our findings above, we hypothesized that Bcl2 may directly regulate Mre11. Because overexpression of Bcl2 did not affect expression levels of Mre11, Rad50 or NBS1 (Figure 4A), we employed co-immunoprecipitation (co-IP) to evaluate the interaction between Bcl2 and Mre11 following treatment of cells with increasing doses (i.e. 0.1–1 Gy) of high-LET IR derived from ^56^Fe or ^28^Si particles. The results of these experiments revealed that high-LET IR exposure promotes the interaction of Bcl2 with Mre11 in a dose-dependent manner (Figure 4B and C). To test whether an increase in Bcl2/Mre11 binding affects DSB repair, we analyzed the percentage of tracks of γH2AX foci remaining at 72 h after treatment of BEAS-2B cells overexpressing WT Bcl2 or vector-only control with increasing doses (0.1, 0.5 and 1 Gy) of ^56^Fe or ^28^Si. Results indicate that the percentage of tracks of γH2AX foci remaining was similar in vector-only control cells following treatment with high-LET IR at various doses. In contrast, the percentage of tracks of γH2AX foci remaining increased in a dose-dependent manner in BEAS-2B cells expressing WT Bcl2 (Supplementary Figure S7). These findings indicate that the dose-dependent increase in Bcl2/Mre11 binding is associated with an increase in the remaining γH2AX foci tracks. These findings reveal that Bcl2/Mre11 binding may negatively regulate the repair of high-LET IR-induced DSBs. Additionally, high-LET IR-enhanced Bcl2/Mre11 binding also resulted in a reduction in Bcl2/Ku70 binding (Figure 4B and C). In contrast, low-LET IR (X-ray) exposure promoted Bcl2 to interact with Ku70 in association with decreased Bcl-2/Mre11 binding (Figure 4D). In a cell-free system, purified Ku70 protein directly disrupted Bcl2/Mre11 complexes *in vitro* (Supplementary Figure S8). These findings suggest a competitive binding of Mre11 and Ku70 with Bcl2, such that the predominant binding partner may be dictated by the nature of the DNA damage stimuli (i.e. high-LET versus low-LET IR). To directly test whether Bcl2 binds to Mre11 or other components (i.e. Rad50 or NBS1) in MRN complexes *in vitro*, purified, recombinant Bcl2 was incubated with purified, recombinant Mre11, Rad50 or NBS1 protein in 1% CHAPS lysis buffer at 4°C for 2 h. Co-IP experiments showed that Bcl2 directly interacts with Mre11 but not Rad50 or NBS1 *in vitro* (Figure 5A). Bcl2 protein contains four BH domains, including BH1, BH2, BH3 and BH4 (35). To determine the binding site of Mre11 on the Bcl2, purified Mre11 protein was incubated with purified recombinant WT or BH deletion Bcl2 mutants in 1% CHAPS lysis buffer. Co-IP experiments showed that Mre11 is able to associate with WT, ΔBH2, ΔBH3, but not with the ΔBH1 and ΔBH4 Bcl2 mutants (Figure 5B), indicating that the BH1 and BH4 domains comprise the Mre11 binding sites on Bcl2.

### Bcl2 disrupts Mre11 complex and inhibits DNA resection

The Mre11 complex is composed of Mre11, Rad50 and NBS1 (i.e. MRN), and is central to the DNA damage response (36). Because Bcl2 can directly interact with Mre11 via its BH1 and BH4 domains (Figures 4 and 5), Bcl2 may affect formation of the functional Mre11 complex via direct interaction with Mre11. To directly test this hypothesis, the Mre11 complex was co-immunoprecipitated from BEAS-2B parental cells. The immune complex was incubated with increasing concentrations of purified, recombinant Bcl2 at 4°C for 1–2 h. The proteins released from the complex were identified in the supernatant following centrifugation. Interestingly, Bcl2 directly disrupted the Mre11 complex *in vitro*, as shown by the decreased levels of bound Rad50 and NBS1 on beads and increased levels of non-bound Rad50 and NBS1 in the supernatant following the addition of purified Bcl2 (Supplementary Figure S9A). To further test whether the Mre11 binding site on Bcl2 is essential for Bcl2 disruption of Mre11 complex, BH deletion mutant proteins were used in similar experiments. Results revealed that the BH1 and BH4 domains are required for the ability of Bcl2 to disrupt the Mre11 complex (Supplementary Figure S9B).

**Figure 6.**
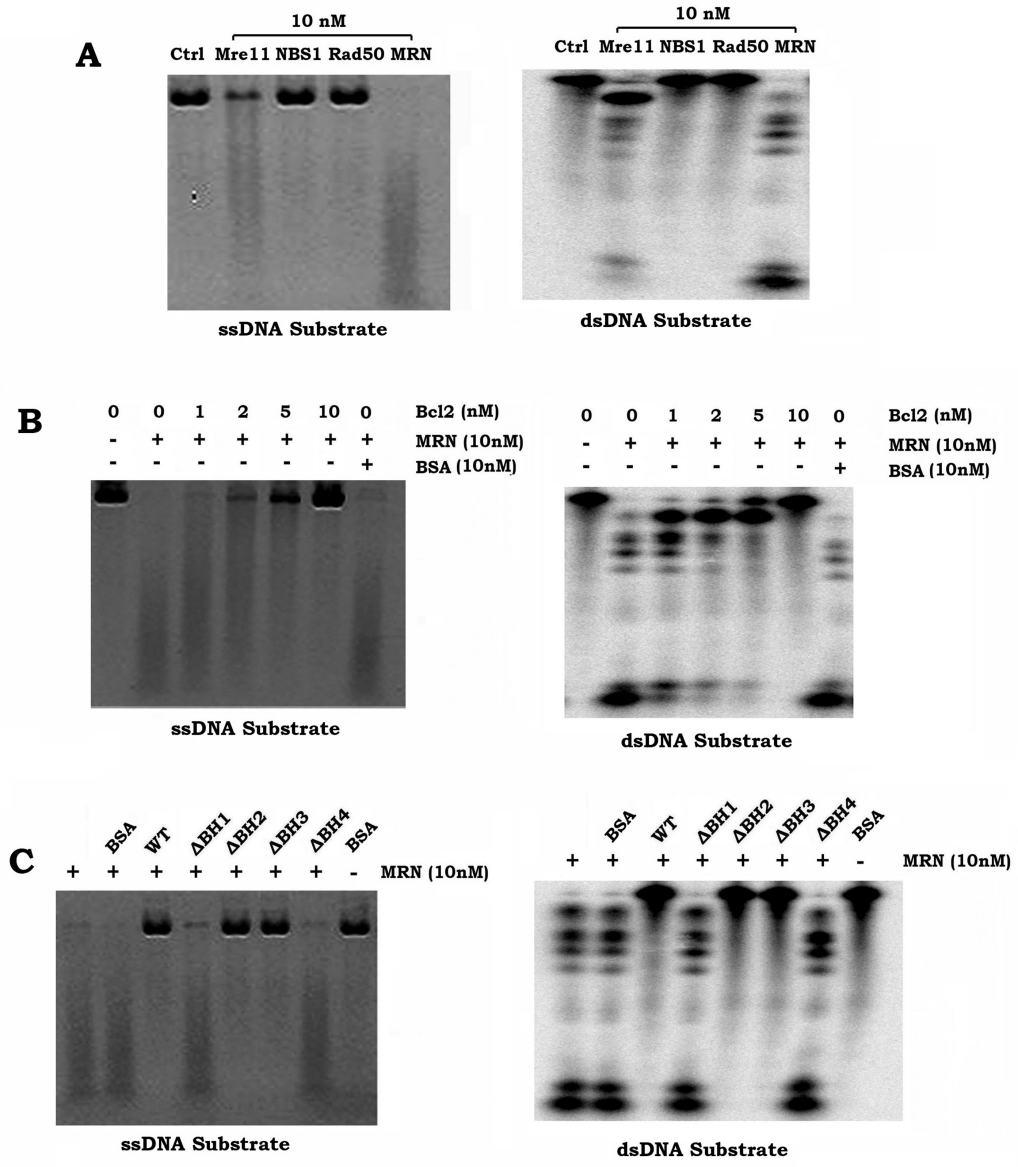
Bcl2 directly inhibits MRN-mediated DNA resection. (**A**) DNA resection activity of purified Mre11, Rad50, NBS1 and MRN complex were measured using PhiX174 circular ssDNA as substrate (left panel: endonuclease activity) and dsDNA as substrate (right panel: exonuclease activity). (**B**) DNA resection activity of MRN complex was measured in the absence or presence of increasing concentrations of purified Bcl2. BSA was used as control. (**C**) DNA resection activity of MRN complex was measured in the absence or presence of increasing concentrations of purified WT Bcl2 or Bcl2 BH deletion mutant protein(s).

DNA end resection is the first and essential step in the repair of high-LET radiation-induced DSBs. To test whether Bcl2 directly regulates DNA resection, we generated purified Mre11, Rad50 and NBS1 proteins from insect cells as described (‘Materials and Methods’ section) (Supplementary Figure S10). We examined DNA resection in a reconstituted system *in vitro* as described (11,19,20). PhiX174 circular single-stranded virion DNA (ssDNA) was used as a substrate for Mre11 endonuclease activity (19). For Mre11 exonuclease activity, double-stranded (ds) DNA substrate was generated by annealing oligonucleotides TP74 to TP124 and labeling with [^32^P] at the 5′ end as described (20). As shown in Figure 6A, single Mre11, but not Rad50 or NBS1, displayed both endonuclease and exonulease activities in DNA resection. The Mre11-Rad50–NSB1 (MRN) complex exhibited greater DNA resection activity as compared to Mre11 alone. Intriguingly, the addition of purified Bcl2 protein directly inhibited MRN-mediated DNA resection in a dose dependent manner (Figure 6B). BSA was used as a control and had no effect on MRN endo- and exonuclease activities. Mre11 binding-deficient Bcl2 protein (i.e. ΔBH1 or ΔBH4) displayed no inhibitory effect on MRN-mediated DNA resection (Figure 6C), suggesting that Mre11 binding is required for the ability of Bcl2 to suppress MRN-mediated DNA resection.

### Bcl2 directly inhibits Mre11/DNA binding *in vitro*

**Figure 7.**
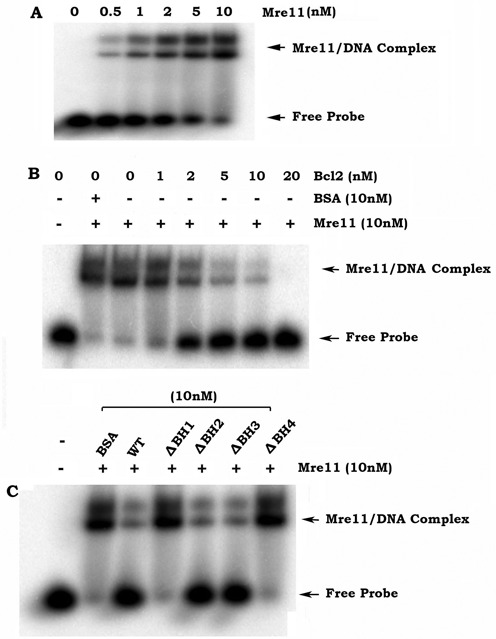
Bcl2 directly inhibits Mre11/DNA binding *in vitro*. (**A**) ^32^P-labelled 3′ overhanging DNA duplexes were incubated with increasing concentrations of Mre11. Mre11/DNA binding was analyzed by EMSA as described in ‘Materials and Methods’ section. (**B**) Effect of increasing concentrations of purified WT Bcl2 protein on Mre11/DNA binding was analyzed by EMSA. (**C**) Effect of various BH deletion Bcl2 mutant proteins on Mre11/DNA binding was analyzed by EMSA.

To test whether Bcl2 directly regulates Mre11/DNA binding, electrophoretic mobility shift assays (EMSA) were carried out in a cell-free system as described (‘Materials and Methods’ section). As shown in Figure 7A, Mre11 bound to the 3′ overhang dsDNA in a dose-dependent manner. The addition of purified Bcl2 protein resulted in a dose-dependent inhibition of Mre11/DNA binding (Figure 7B). Intriguingly, removal of the Mre11 binding site (BH1 or BH4) resulted in failure of Bcl2 to suppress Mre11/DNA binding (Figure 7C), suggesting that the Mre11 binding site is essential for Bcl2 to directly disrupt the Mre11/DNA complex.

### BH1 and BH4 domains are required for the inhibitory effects of Bcl2 on Mre11/DNA binding and DSB repair following exposure to high-LET IR

To further test the role of Bcl2/Mre11 binding, BEAS-2B cells expressing WT Bcl2 or various BH domain deletion mutants were exposed to 1 Gy of ^56^Fe or ^28^Si particles. HR activity, Mre11/DNA binding and DSB tracks were analyzed. Removal of the BH1 or BH4 from Bcl2 eliminated its inhibitory effects on HR activity and high-LET IR-induced Mre11/DNA binding (Supplementary Figure S11). In contrast, deletion of the BH2 or BH3 domain did not affect the ability of Bcl2 to suppress HR activity and Mre11 DNA binding (Supplementary Figure S11). Deletion of either BH1 or BH4 domain from Bcl2 resulted in failure of Bcl2 to inhibit the repair of high-LET IR-induced DSBs as determined by γH2AX tracks (Supplementary Figures S12 and S13). These findings support the notion that the binding of Bcl2 to Mre11 via the BH1 or BH4 domain is required for Bcl2 suppression of HR activity, high-LET IR-induced Mre11/DNA binding and the repair of DSBs induced by high-LET IR.

### Depletion of endogenous Bcl2 by shRNA enhances HR activity and high-LET IR-induced Mre11 DNA binding leading to acceleration of repair of high-LET IR-induced DSBs

To analyze the role of Bcl2 at physiological levels in regulating HR activity, Mre11/DNA binding and the repair of DSBs, endogenous Bcl2 was depleted using Bcl2 shRNA from H460 cells expressing high levels of endogenous Bcl2 (Supplementary Figure S14A). HR activity, Mre11/DNA binding and γ-H2AX tracks were compared in H460 cells expressing Bcl2 shRNA or control shRNA in the absence or presence of high-LET IR exposure from ^56^Fe or ^28^Si particles. The results revealed that knockdown of endogenous Bcl2 resulted in up-regulation of HR activity and enhanced high-LET IR-induced Mre11/DNA binding in association with accelerated disappearance of γ-H2AX foci track (Supplementary Figures S14 and S15). These findings strongly suggest that physiologically expressed Bcl2 in cells is able to suppress DSB repair by a mechanism involving the HR pathway following high-LET IR exposure.

## DISCUSSION

High-LET IR derived from HZE particles (i.e. ^56^Fe or ^28^Si) possesses both high energy and high mass, producing densely spaced clustered DSBs along the ion track (Figures 1 and 2). In contrast, the damage caused by low-LET IR (i.e. X-rays) is much more widely spaced due to the lower energy and larger distances between ionization events, with DNA breaks scattered more evenly throughout the nucleus (Figure 1) (37). Although both high- and low-LET IR produce similar DSB numbers, the closely packed DSBs caused by high-LET IR are more likely to result in the generation of small double stranded DNA (dsDNA) fragments compared to low-LET IR (38,39). NHEJ often fails to contribute to the repair of high-LET IR-induced DSBs, which is more dependent upon HR repair pathways (37).

Bcl2, a major antiapoptotic and oncogenic protein, has been previously shown to retard the repair of low-LET IR-induced DSBs via inhibition of the NHEJ pathway (13). Here we demonstrate that Bcl2 can also inhibit the repair of high-LET IR-induced DNA damage via suppression of the HR pathway (Figures 2 and 3). It is known that Mre11 complex-mediated DNA resection is an essential step for initiating the HR DNA repair pathway (32–34). Our findings indicate that Bcl2 not only inhibits Mre11 binding to chromatin DNA in cells following high-LET IR exposure but also directly suppresses Mre11 binding to the 3′ overhang dsDNA in a cell-free system (Figures 3 and 7), suggesting that Bcl2 may interfere with recruitment of Mre11 to the DSB site where it exerts its DNA resection activity.

Because high-LET IR derived from ^56^Fe or ^28^Si enhances Bcl2/Mre11 binding but not Bcl2/Ku70 binding (Figure 4B and C), this suggests that Bcl2 may only inhibit the HR but not NHEJ repair pathway following exposure of high-LET IR. In contrast, low-LET IR derived from X-rays enhances Bcl2/Ku70 binding but not Bcl2/Mre11 binding (Figure 4D), suggesting that Bcl2 may only inhibit the NHEJ but not HR repair pathway following low-LET IR. We have previously demonstrated that Bcl2 inhibits the Ku-dependent NHEJ DNA repair pathway following low-LET radiation (13). Based on our current findings, Bcl2 may have different responses to low-LET versus high-LET IR in the choice of its binding partner (Ku70 or Mre11) and in which DSB repair pathway (NHEJ or HR) it negatively regulates. Mre11 complex-mediated DNA resection is the initial step, which is required for Rad51 to initiate the HR repair pathway (11). Since Bcl2 can negatively regulate the Rad51-dependent HR repair pathway (14), it is possible that, in addition to the DNA resection stage, Bcl2 may also inhibit Rad51 in the HR repair pathway following high-LET radiation. Further work is required to test this hypothesis.

Recent reports reveal that high-LET IR derived from HZE particles not only induces DSBs but also generates small DNA fragments. In contrast, low-LET IR only induces DSBs without the generation of small DNA fragments (2,3). High-LET IR-induced small DNA fragments have been reported to potently inhibit the NHEJ pathway but do not affect the HR repair pathway (3,24,27), indicating that the HR but not the NHEJ pathway plays a major role in repairing high-LET radiation-induced DSBs. Although high-LET IR does not stimulate, and even slightly reduces, Bcl2/Ku70 association (Figure 4B and C), NHEJ may still not be active because high-LET IR-induced small DNA fragments are able to inhibit NHEJ, as recently reported (2,3).

Subunit and domain mapping reveal that Bcl2 directly interacts with Mre11 but not Rad50 or NBS1 via its BH1 and BH4 domains (Figure 5). Since Ku70 has also been previously found to interact with Bcl2 at the BH1 and BH4 domains (13), this suggests that Ku and Mre11 bind to Bcl2 at the same site. These findings help explain why Mre11 and Ku70 compete with each other for Bcl2 binding (Figure 4 and Supplementary Figure S8).

The MRN complex promotes DNA end resection through at least three different mechanisms, including 5′ strand endonucleolytic degradation by Mre11, recruitment of other enzymes and displacement of the Ku heterodimer that promotes NHEJ (40). Because Mre11 binding to DNA ends is the first step in its DNA resection function and subsequently its activation of the HR pathway (32–34), Bcl2 binding to Mre11 may interfere with MRN complex-mediated DNA end resection. Here, we demonstrate that Bcl2 not only dissociates Rad50 and NBS1 from the MRN complex via its Mre11 binding (Supplementary Figure S9A), but also directly suppresses MRN-mediated DNA resection activity (Figure 6B). Importantly, the Mre11 binding sites on Bcl2 (i.e. BH1 and BH4 domains) are required for the ability of Bcl2 to disrupt the MRN complex as well as for its inhibitory effect on DNA resection (Figure 6C and Supplementary Figure S9B). Additionally, Mre11 binding deficient Bcl2 mutants (i.e. ΔBH1 and ΔBH4) lose their abilities to inhibit HR, Mre11/DNA binding and the repair of clustered DSBs following high-LET IR (Supplementary Figures S11, S12 and S13). Based on these findings, we conclude that the inhibitory effect of Bcl2 on HR-mediated repair of high-LET IR-induced clustered DSBs may occur through a mechanism that involves inhibition of DNA resection via direct interaction with Mre11.

Mre11 has endonuclease activity that promotes 5′-3′ resection of DNA ends, as well as 3′-5′ exonuclease activity (33). Our findings confirm that Mre11 has both endo- and exonuclease activities (Figure 6A), which may trim DNA ends for repair. Intriguingly, Mre11 can also cause degradation of stalled DNA replication forks, leading to nascent strand shortening (41,42). It is possible that the inhibitory effect of Bcl2 on Mre11 may alleviate degradation of stalled forks. The replication fork protective role of Bcl2 may oppose its repair inhibition effect. However, we recently discovered that Bcl2 also suppresses ribonucleotide reductase activity via direct interaction with hRRM2, leading to decreased intracellular dNTP and retardation of DNA replication fork progression (43). Thus, Bcl2 may have a dual role in fork progression by regulating Mre11 or hRRM2. It is possible that the inhibitory effect of Bcl2 on ribonucleotide reductase may overrule its fork protection activity from Mre11 inhibition.

High-LET IR-induced clustered DSBs are genotoxic DNA lesions that, if unrepaired or repaired incorrectly, can lead to mutations, chromosomal breaks or translocations with malignant transformation (37,44). Mutagenesis following high-LET IR is further exacerbated as the rejoining of clustered DSBs to one another is more likely to be aberrant due to the extreme proximity of multiple DSB ends (45). Bcl2 has been previously reported to have two major functions, including promoting cell survival and inhibition of DSB repair (13). Based on our findings, we propose that, after exposure of cells to ^56^Fe or ^28^Si to induce clustered DSBs, the inhibitory effect of Bcl2 on clustered DSB repair, plus its role in supporting survival may result in surviving cells with accumulated unrepaired or partially clustered DSBs, which may further contribute to the enhancement of HZE particle-induced malignant transformation.

In summary, this study demonstrates that suppression of clustered DSB repair by Bcl2 occurs through inhibition of DNA resection and HR. HZE particle-induced clustered DSBs stimulate Bcl2 to associate with Mre11. Direct interaction of Bcl2 with Mre11 via its BH1 and BH4 domains may be required for Bcl2 to inhibit Mre11 DNA binding, formation of MRN complexes, and HR activity, which lead to suppression of the repair of high-LET IR-induced clustered DSBs. Therefore, Bcl2, in addition to its antiapoptotic properties, may also function as an oncogenic molecule to inhibit the repair of HZE particle-induced clustered DSBs through the HR pathway.

## SUPPLEMENTARY DATA

Supplementary Data are available at NAR Online.

SUPPLEMENTARY DATA
