# Supplementary material for: Bcl2 inhibits recruitment of Mre11 complex to DNA double-strand breaks in response to high-linear energy transfer radiation
Source: Nucleic Acids Res. 2015 Jan 7;43(2):960–72. doi: 10.1093/nar/gku1358 (PMC4333404; doi:10.1093/nar/gku1358)
Supplement: SUPPLEMENTARY DATA [file supp_43_2_960__index.html]

Bcl2 inhibits recruitment of Mre11 complex to DNA double-strand breaks in response to high-linear energy transfer radiation — SUPPLEMENTARY DATA 

# Bcl2 inhibits recruitment of Mre11 complex to DNA double-strand breaks in response to high-linear energy transfer radiation

## SUPPLEMENTARY DATA

**Files in this Data Supplement:**

- SUPPLEMENTARY DATA
