# Supplementary material for: Bcl2 inhibits recruitment of Mre11 complex to DNA double-strand breaks in response to high-linear energy transfer radiation
Source: Nucleic Acids Res. 2015 Jan 7;43(2):960–72. doi: 10.1093/nar/gku1358 (PMC4333404; doi:10.1093/nar/gku1358)
Supplement: SUPPLEMENTARY DATA [file supp_gku1358_nar-02100-d-2014-File009.pdf]

Supplementary Data

Bcl2 inhibits recruitment of Mre11 complex to DNA double-strand breaks in response to high-linear energy transfer radiation

Maohua Xie, Dongkyoo Park, Shuo You, Rui Li, Taofeek K. Owonikoko, Ya Wang, Paul W. Doetsch, and Xingming Deng

Inventory of Supplementary Information

- **Supplementary Data**

Supplementary Figure S1
Supplementary Figure S2
Supplementary Figure S3
Supplementary Figure S4
Supplementary Figure S5
Supplementary Figure S6
Supplementary Figure S7
Supplementary Figure S8
Supplementary Figure S9
Supplementary Figure S10
Supplementary Figure S11
Supplementary Figure S12
Supplementary Figure S13
Supplementary Figure S14
Supplementary Figure S15

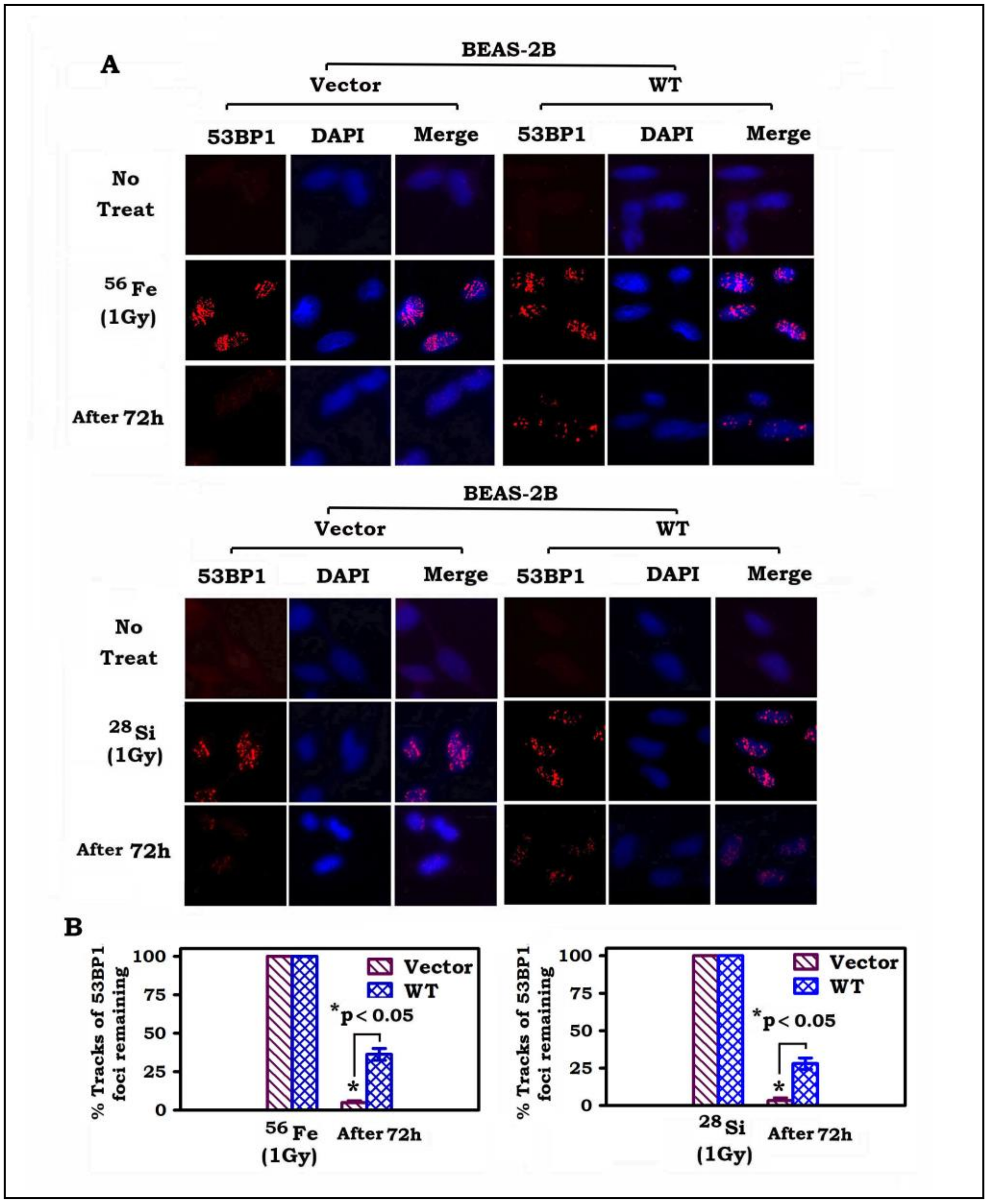

Supplementary Figure S1. Expression of Bcl2 retards the repair of high-LET IR-induced clustered DSBs. (**A**) BEAS-2B cells overexpressing Bcl2 or vector-only control were exposed to 1 Gy of ^{56}Fe or ^{28}Si. Cells were harvested after 72h. DSB tracks were measured by immunostaining using 53BP1 antibody. (**B**) 53BP1 tracks were quantified as described in "Methods".

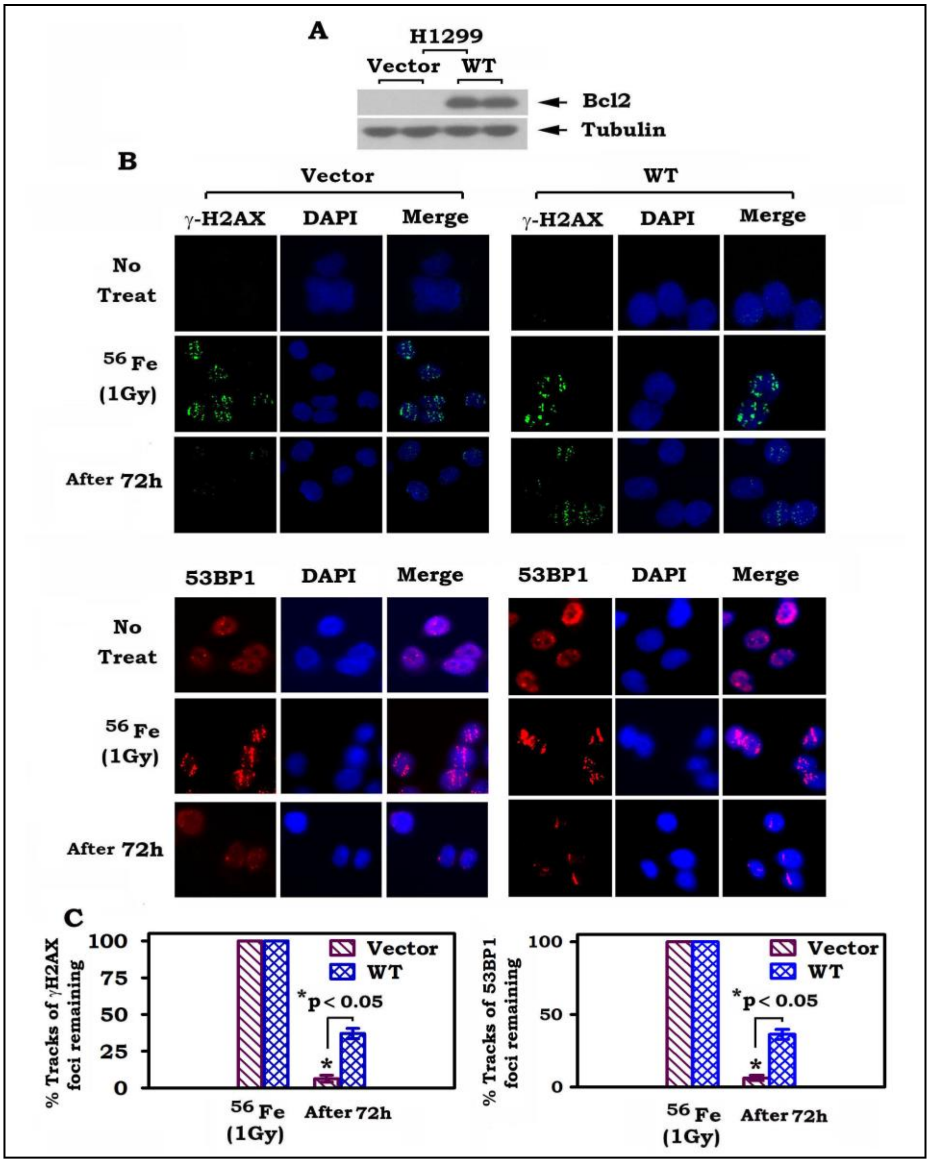

Supplementary Figure S2. Expression of Bcl2 retards the repair of high-LET IR (^{56}Fe)-induced clustered DSBs in H1299 cells. (**A**) Bcl2 expression was analyzed by Western blot in H1299 cells overexpressing WT Bcl2 or vector-only control. (**B**) H1299 cells overexpressing Bcl2 or vector-only control were exposed to 1 Gy of ^{56}Fe. Cells were harvested after 72h. DSB tracks were detected by immunostaining using γ-H2AX or 53BP1 antibody, respectively. (**C**) γ-H2AX or 53BP1 tracks were quantified as described in “Methods”.

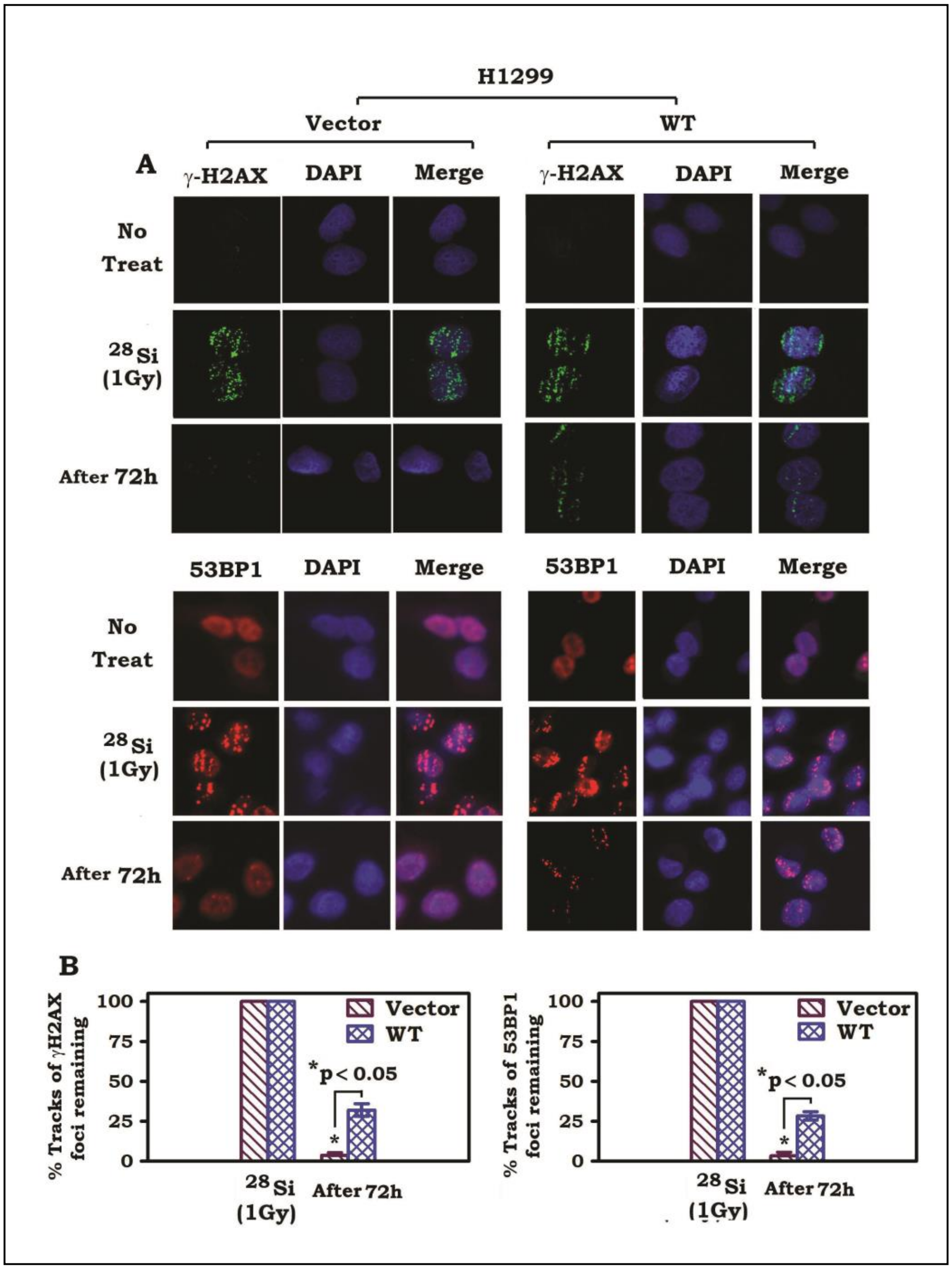

Supplementary Figure S3. Expression of Bcl2 retards the repair of high-LET IR (^{28}Si)-induced clustered DSBs in H1299 cells. (**A**) H1299 cells overexpressing Bcl2 or vector-only control were exposed to 1 Gy of ^{28}Si. Cells were harvested after 72h. DSB tracks were detected by immunostaining using γ-H2AX or 53BP1 antibody, respectively. (**B**) γ-H2AX or 53BP1 tracks were quantified as described in “Methods”.

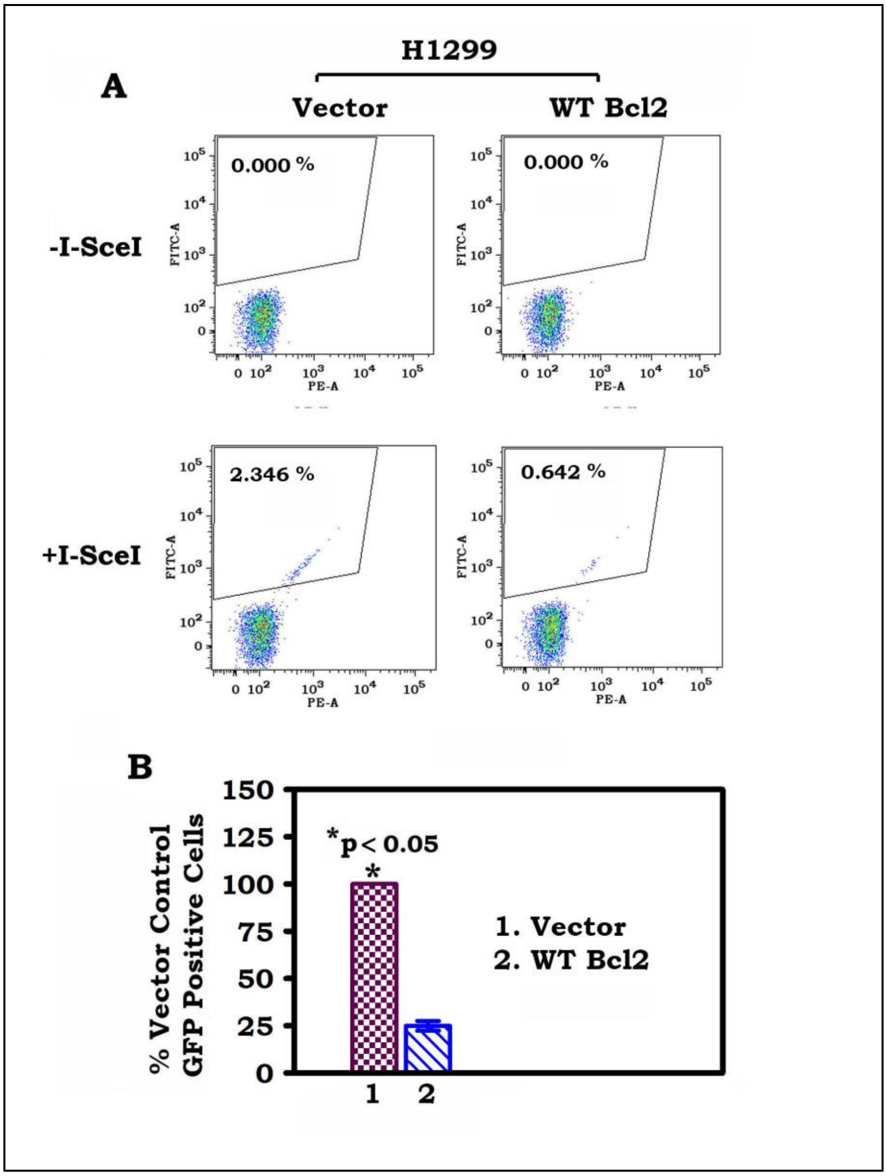

Supplementary Figure S4. Expression of Bcl2 suppresses homologous recombination (HR) in H1299 cells. HR activity was measured using pCBASce and DR-GFP system in H1299 cells overexpressing Bcl2 or vector-only control as described in “Methods”. Percentage of GFP-positive cells was analyzed by flow cytometry.

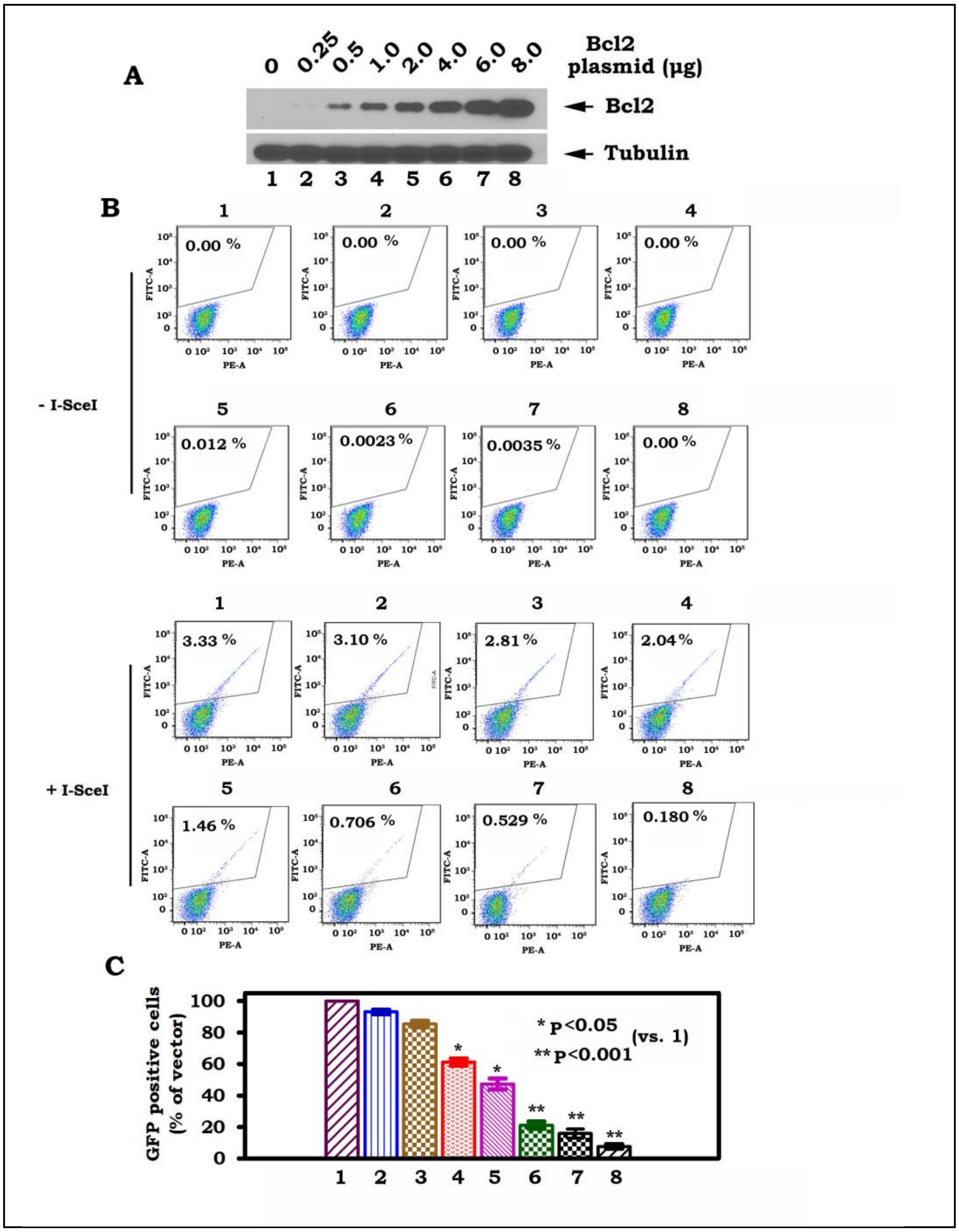

Supplementary Figure S5. Expression of various levels of Bcl2 in BEAS-2B cells results in dose-dependent suppression of homologous recombination (HR). (A) Various amounts of Bcl2 cDNA in pCIneo mammalian expression vector were transfected into BEAS-2B cells. Expression levels of Bcl2 were analyzed by Western blot. (B) and (C) HR activity was measured using pCBASce and DR-GFP system in BEAS-2B cells expressing various levels of Bcl2 as described in “Methods”. Percentage of GFP-positive cells was analyzed by flow cytometry.

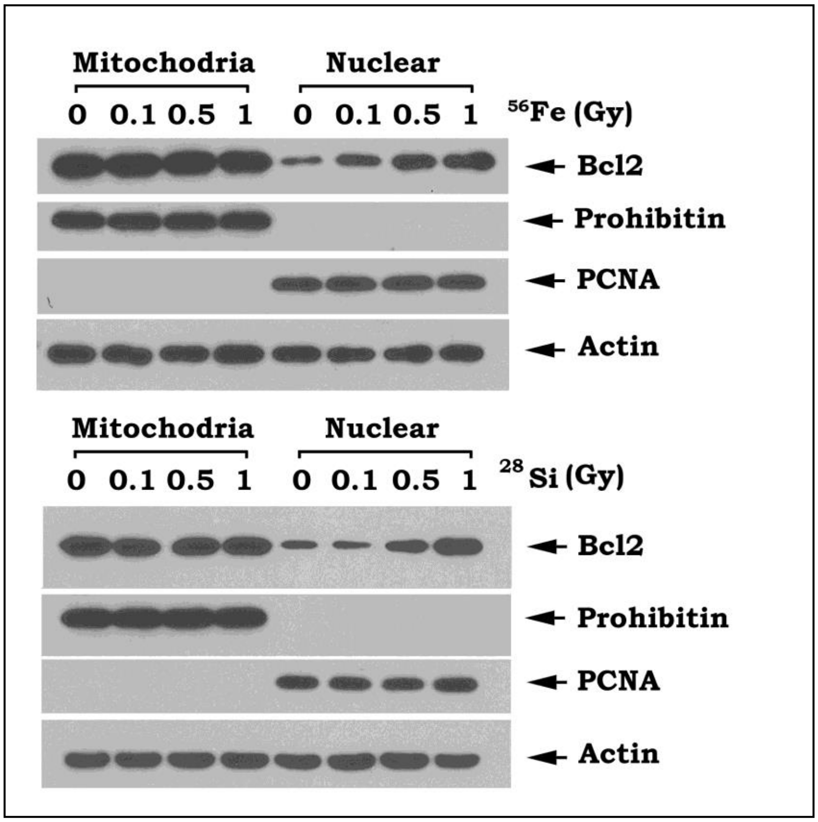

Supplementary Figure S6. High-LET radiation derived from ^{56}Fe and ^{28}Si particles stimulates Bcl2 accumulation in nucleus**.** BEAS-2B cells expressing WT Bcl2 were treated with increasing doses of high-LET radiation derived from ^{56}Fe and ^{28}Si particles. Subcellular fractionation was performed to isolate nuclear (Nuc) and mitochondrial (Mito) fractions. Western blot analysis of subcellular fractions was performed to detect Bcl2. Prohibitin or PCNA was used as a mitochondrial or nuclear marker, respectively.

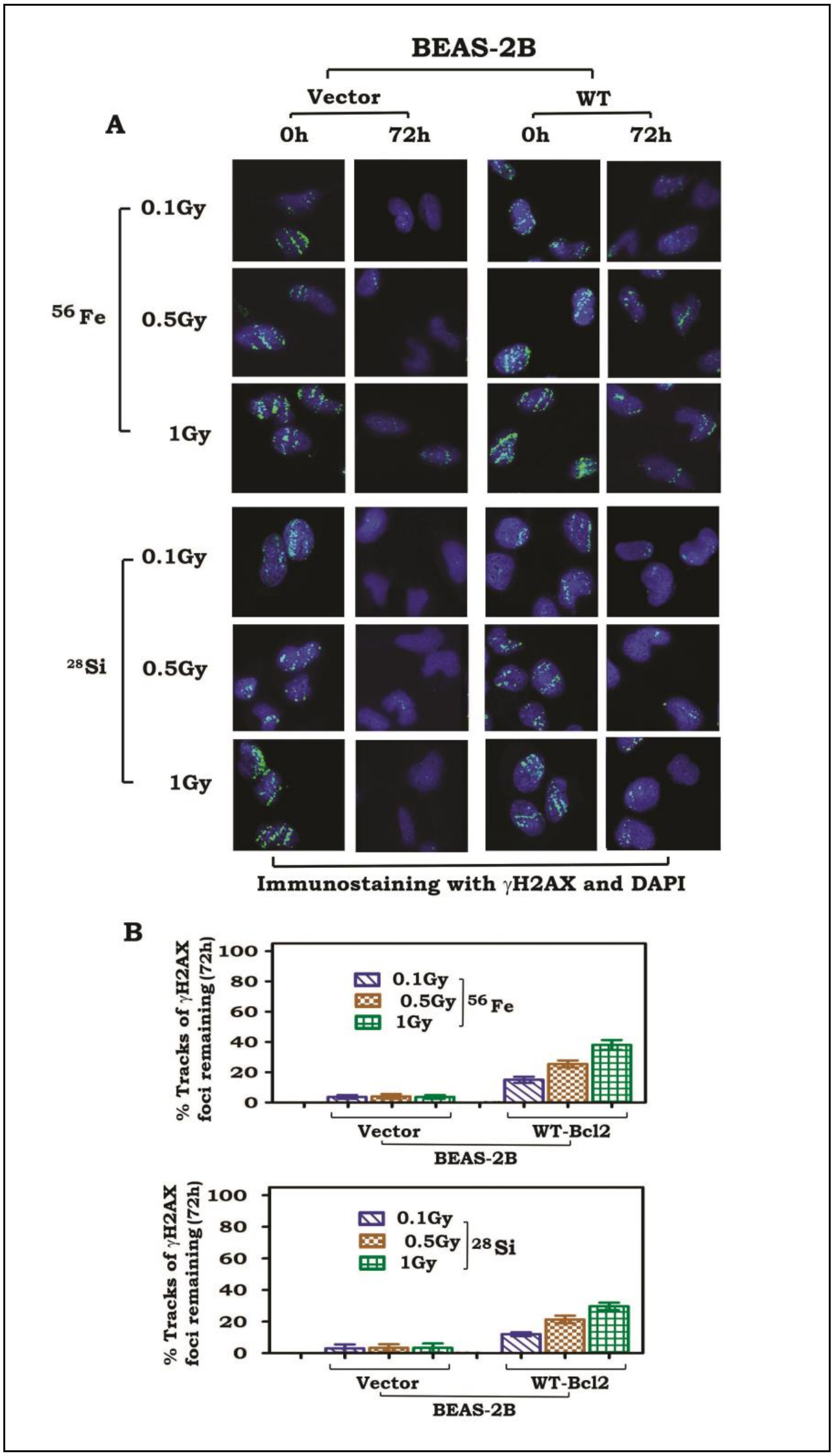

Supplementary Figure S7. Expression of Bcl2 retards DSB repair following increasing doses of high-LET radiation derived from ^{56}Fe and ^{28}Si particles. (**A**) BEAS-2B cells overexpressing WT-Bcl2 or vector-only control were treated with increasing doses of high-LET radiation derived from ^{56}Fe and ^{28}Si particles. Cells were harvested at 0h and 72h. DSB tracks were measured by immunostaining using γ-H2AX and DAPI antibodies. (**B**) γ-H2AX foci tracks were quantified as described in “Methods”.

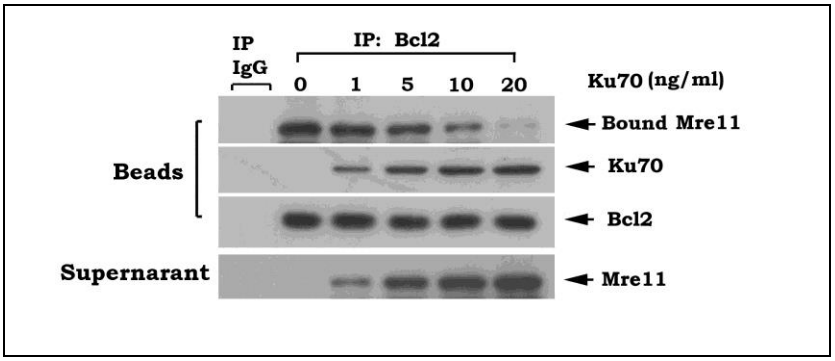

Supplementary Figure S8. Purified Ku70 protein disrupts Bcl2/Mre11 complex. Purified Bcl2 was incubated with purified Mre11 in 1% CHAPS lysis buffer at 4℃ for 2h. Bcl2/Mre11 complex was immunoprecipitated using agarose-conjugated Bcl2 antibody. The immuno-complexes were then incubated with increasing concentrations of Ku70 protein in 1% CHAPS lysis buffer at 4℃ for 2h. After centrifugation, the immunocomplexes on beads and supernatant were subjected to SDS-PAGE and analyzed by Western blot using Mre11, Ku70 or Bcl2 antibody, respectively.

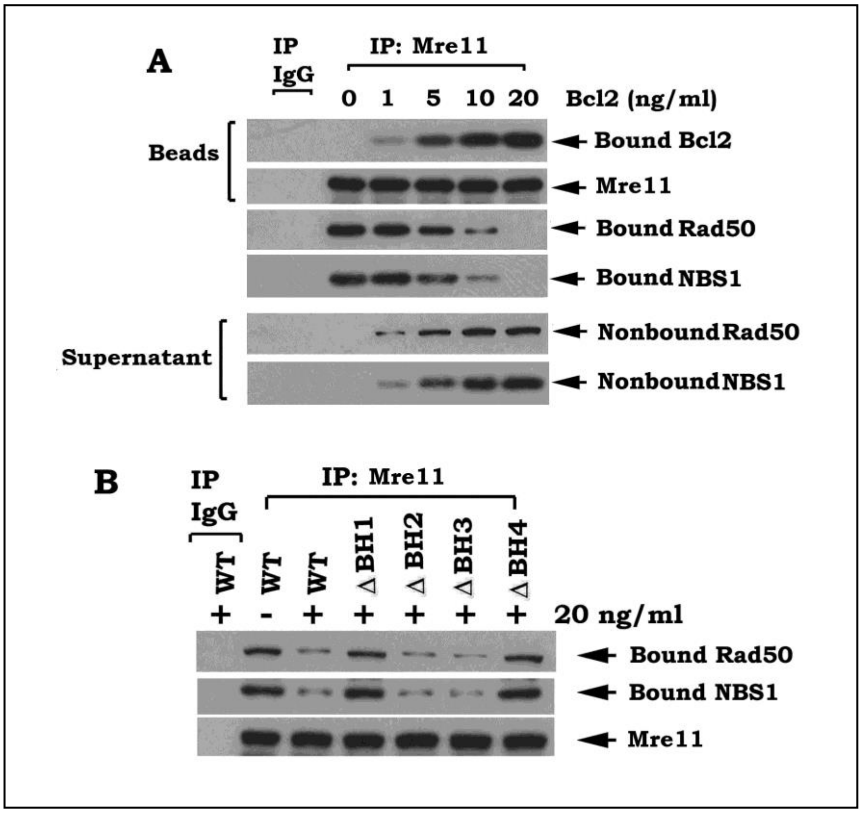

Supplementary Figure S9. Bcl2 directly disrupts the MRN complex, which requires the BH1 and BH4 domain. (**A**) MRN complex was coimmunoprecipitated from BEAS2B cells using Mre11 antibody, and then incubated with increasing concentrations of purified Bcl2 protein in 1% CHAPS lysis buffer at 4^oC for 2 hours. After centrifugation, the resulting supernatant or immunocomplexed beads were subjected to SDS-PAGE. Bcl2, Mre11, RAD50 and NBS1 on beads or in supernatant were analyzed by Western blot. (**B**) The MRN complex was obtained as above and incubated with purified WT or each of the BH deletion Bcl2 mutant proteins in 1% CHAPS lysis buffer at 4^oC for 2 hours. After centrifugation and washing, the immunocomplexes on beads were subjected to SDS-PAGE and analyzed by Western blot to measure Mre11-associated Rad50 and NBS1.

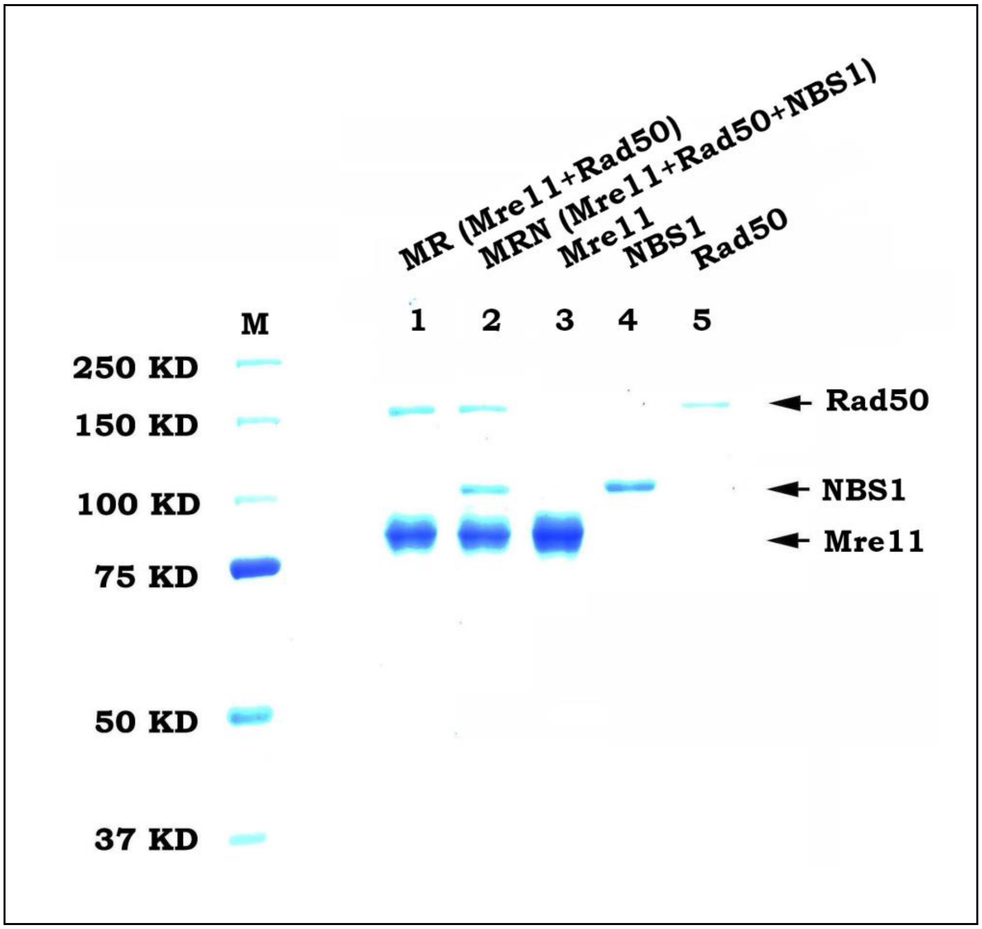

Supplementary Figure S10. Production and purification of MRN. The MRN complex as well as the individual MRN subunits (Mre11, Rad50 and NBS1) were produced and purified from Sf9 cells. Purified proteins were subjected to SDS-PAGE and stained by Coomassie blue.

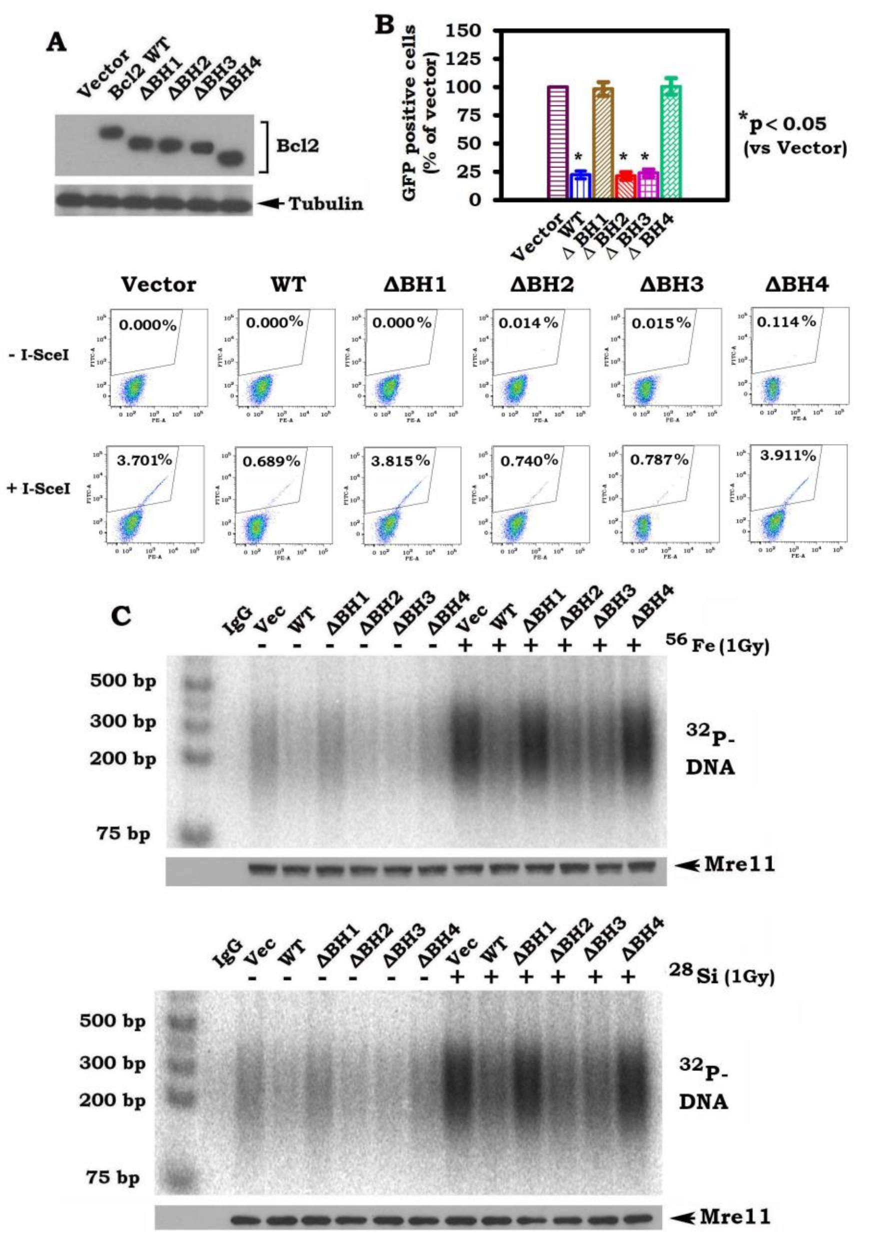

Supplementary Figure S11. BH1 and BH4 domains are essential for Bcl2 to suppress high-LET-induced Mre11/DNA binding. (**A**) WT or each of the BH deletion Bcl2 mutants were stably transfected into BEAS-2B cells. Expression of Bcl2 was analyzed by Western blot. (**B**) HR activity was measured in BEAS-2B cells expressing WT or each of the BH deletion Bcl2 mutants as described in “Methods”. Percentage of GFP-positive cells was analyzed by flow cytometry. Error bars represent ± SD of three repeated determinations. (**C**) BEAS-2B cells expressing WT or each of the BH deletion Bcl2 mutants were exposed to 1 Gy of ^{56}Fe or ^{28}Si particles. Mre11/DNA binding was analyzed by ChIP assay.

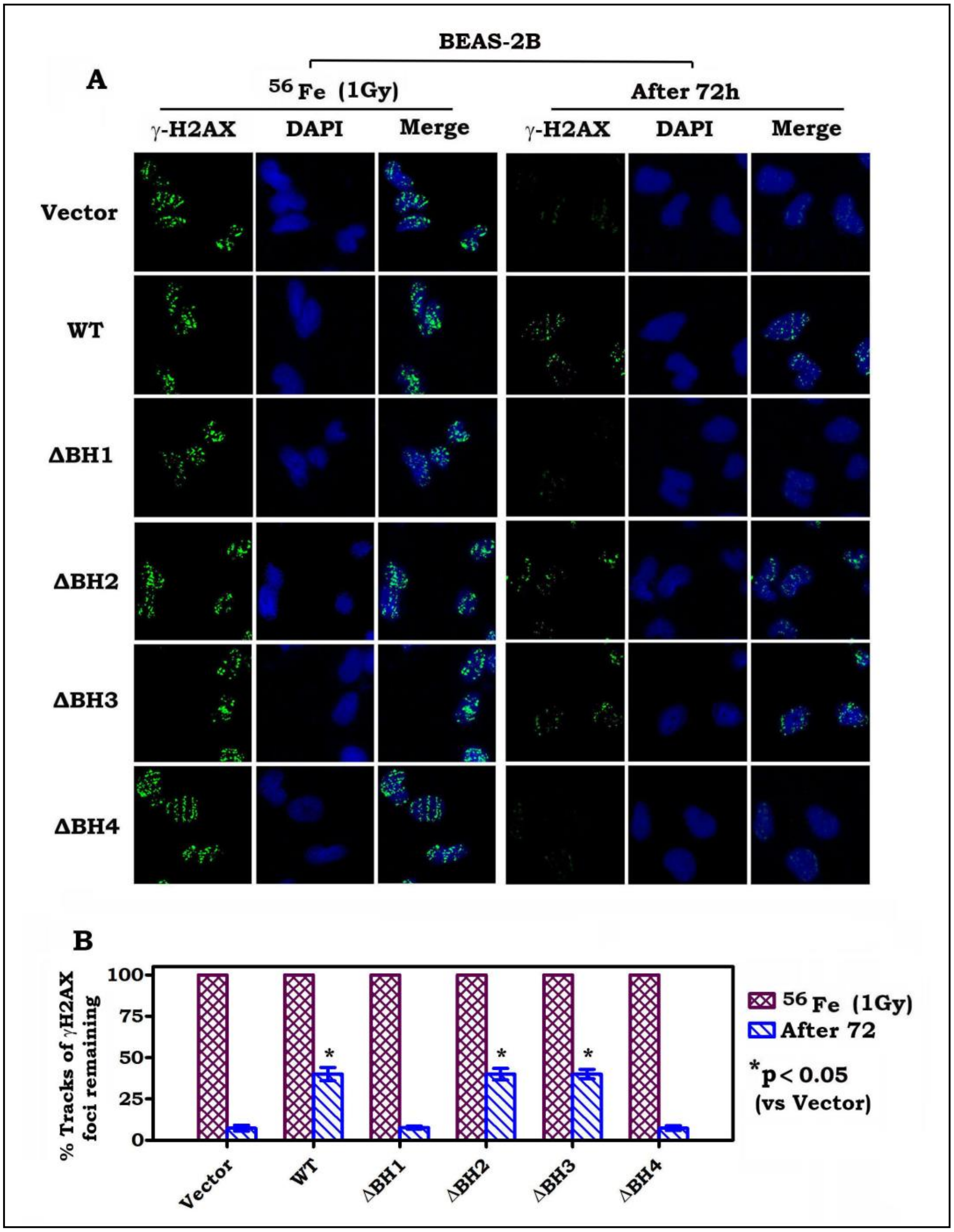

Supplementary Figure S12. BH1 and BH4 domains are essential for Bcl2 to suppress DSB repair following exposure to ^{56}Fe particles. (**A**) BEAS-2B cells expressing WT or each of the BH deletion Bcl2 mutants were exposed to 1 Gy of ^{56}Fe particles. Cells were harvested after 72h. DSB tracks were measured by immunostaining using γ-H2AX antibody. (**B**) γ-H2AX foci tracks were quantified as described in "Methods".

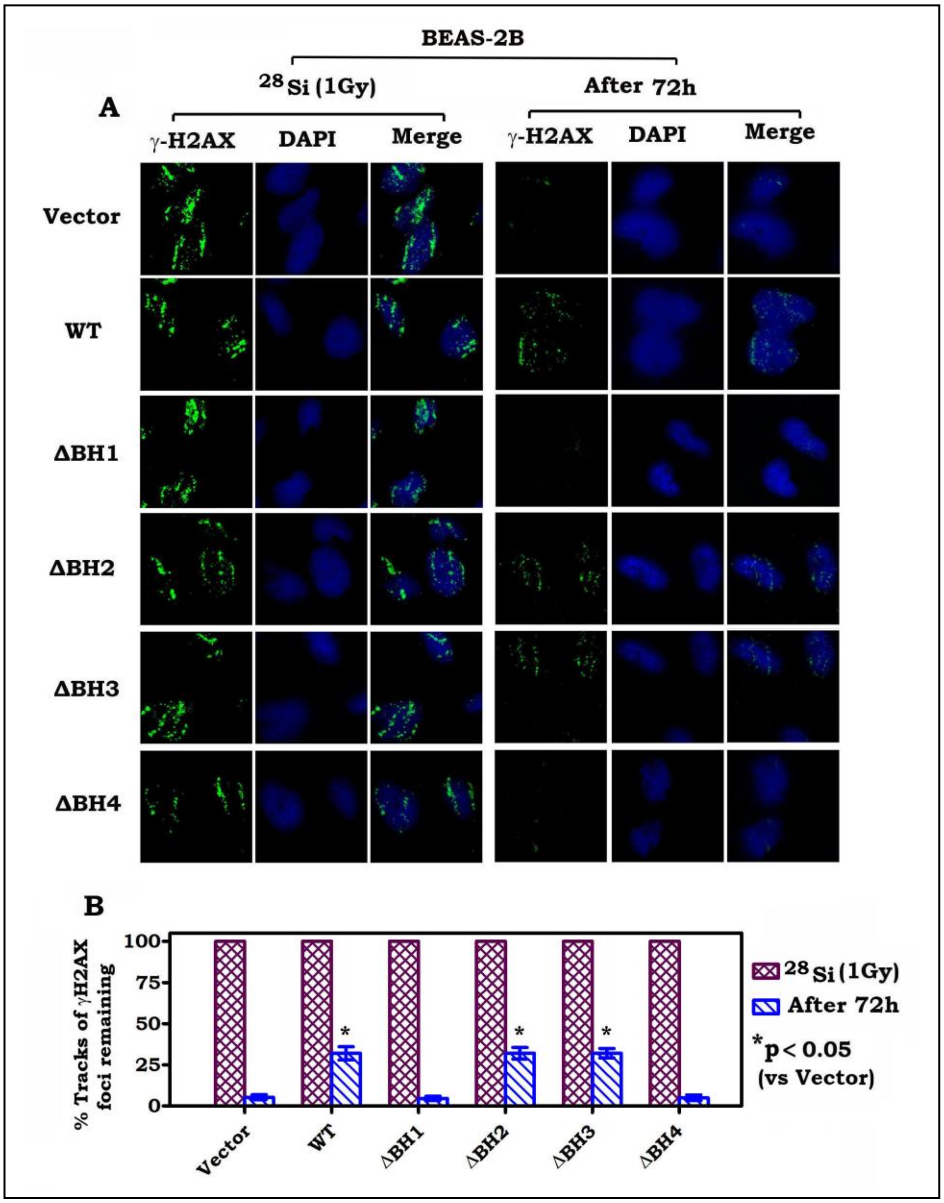

Supplementary Figure S13. BH1 and BH4 domains are essential for Bcl2 to suppress DSB repair following exposure to ^{28}Si particles. (**A**) BEAS-2B cells expressing WT or each of the BH deletion Bcl2 mutants were exposed to 1 Gy of ^{28}Si particles. Cells were harvested after 72h. DSB tracks were measured by immunostaining using γ-H2AX antibody. (**B**) γ-H2AX foci tracks were quantified as described in "Methods".

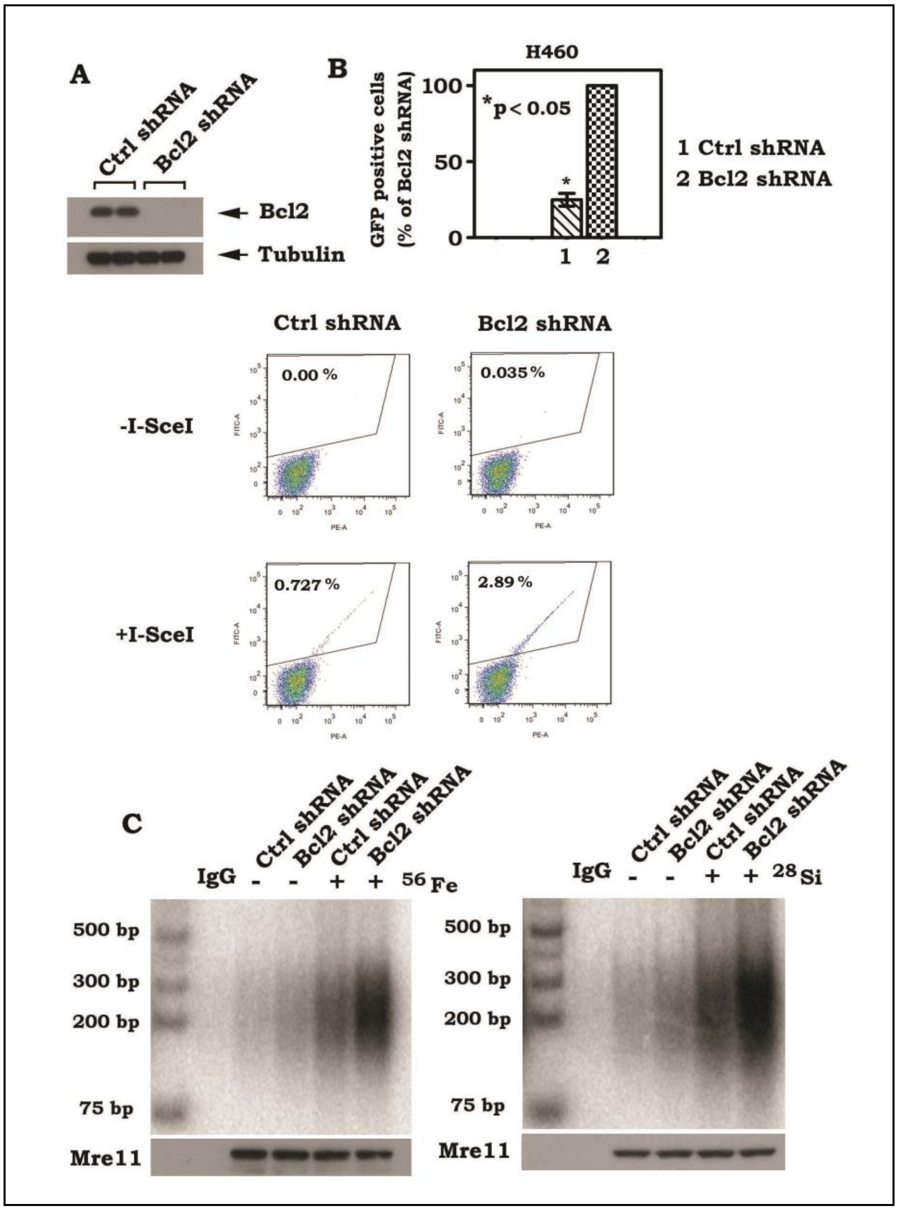

Supplementary Figure S14. Depletion of endogenous Bcl2 results in up-regulation of HR activity and enhanced Mre11/DNA binding. (**A**) Bcl2 shRNA or control shRNA was stably transfected in H460 cells that express high levels of endogenous Bcl2. Expression levels of Bcl2 were analyzed by Western blot analysis. (**B**) HR activity was measured in H460 cells expressing Bcl2 shRNA or control shRNA. (**C**) H460 cells expressing Bcl2 shRNA or control shRNA were exposed to 1 Gy of ^{56}Fe or ^{28}Si particles. Mre11/DNA binding was analyzed by ChIP assay.

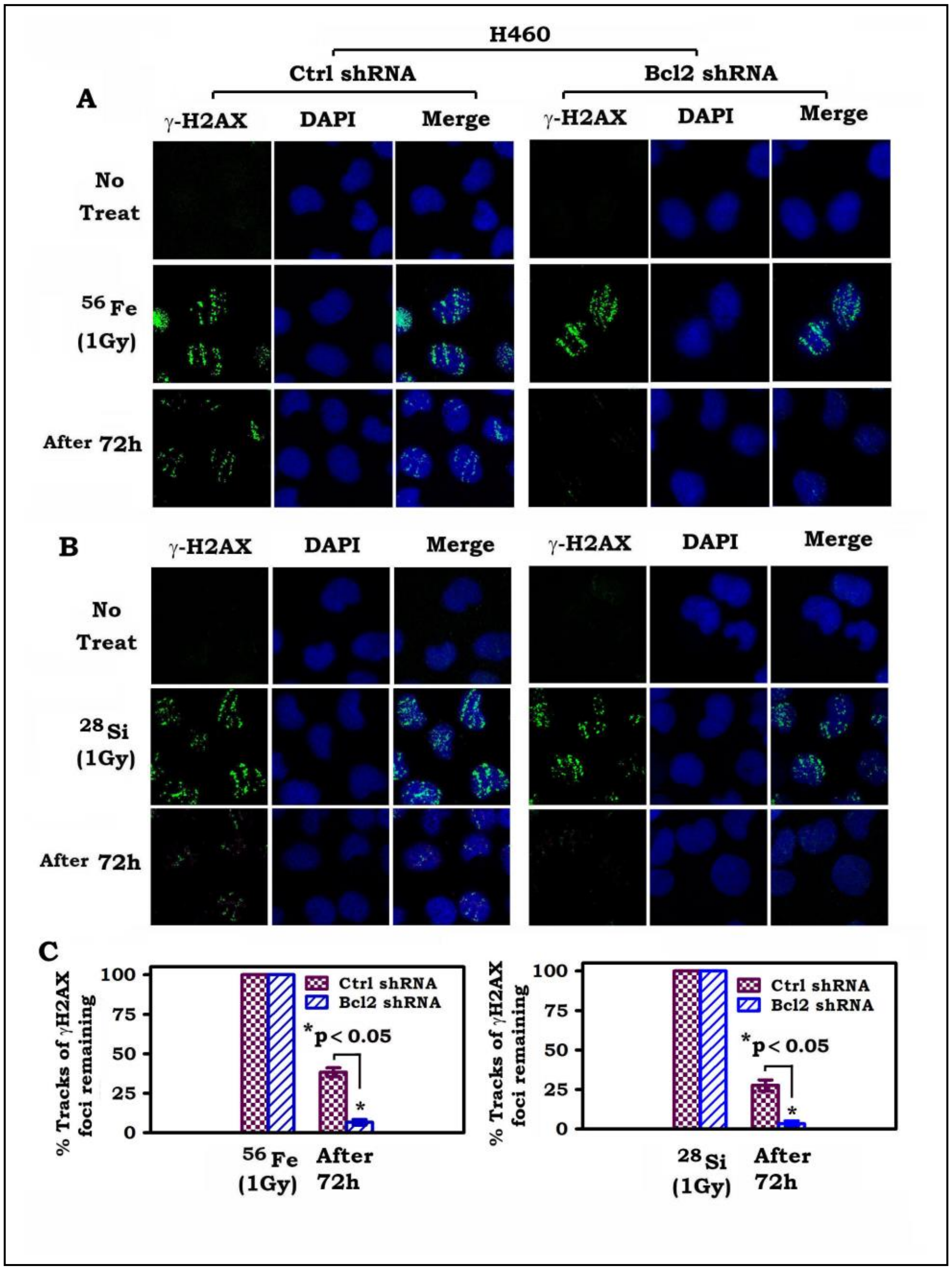

Supplementary Figure S15. Depletion of endogenous Bcl2 results in accelerated repair of high-LET-induced clustered DSBs. (**A**) and (**B**) H460 cells expressing Bcl2 shRNA or control shRNA were exposed to 1 Gy of ^{56}Fe or ^{28}Si particles. Cells were harvested after 72h. DSB tracks were measured by immunostaining using γ-H2AX antibody. (**C**) γ-H2AX foci tracks were quantified as described in "Methods".
